# Total Synthesis and Antidepressant Activities of Laetispicine and Its Derivatives

**DOI:** 10.3390/molecules17021425

**Published:** 2012-02-03

**Authors:** Shuyi Yao, Hui Xie, Li Zhang, Tao Meng, Yongliang Zhang, Xin Wang, Lin Chen, Shengli Pan, Jingkang Shen

**Affiliations:** 1 State Key Laboratory of Drug Research, Shanghai Institute of Materia Medica, Chinese Academy of Sciences, 555 Zuchongzhi Road, Shanghai 201203, China; 2 School of Pharmacy, Fudan University, 826 Zhangheng Road, Shanghai 201203, China

**Keywords:** laetispicine derivatives, antidepressant, total synthesis, forced swimming test

## Abstract

The first total synthesis of laetispicine (**1a**), an amide alkaloid isolated from the stems of *Piper laetispicum* C.DC (Piperaceae), and the synthesis of some of its derivatives were described. Based on the evaluation of antidepressant activities in the forced swimming test, compounds **1h** and **1i** were identified as potent and safe antidepressant lead compounds.

## 1. Introduction

**Figure 1:**
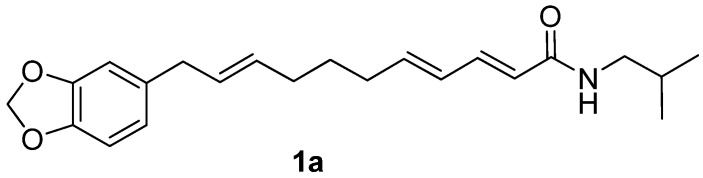
Structure of laetispicine.

Natural products have traditionally played an important role in drug discovery and are the basis of many important therapeutics that have found broad use in the clinic [1]. Extensive studies to search among natural products for new antidepressants that possess both high efficacy and safety have been carried out [2,3]. Laetispicine (Figure 1) was first isolated in 2002 by Pan and co-workers from *Piper laetispicum* C.DC., a herb growing in China and parts of southeast Asia [4,5]. *Piper laetispicum* C.DC. is one of the species in pepper family and had been used for invigorating circulation and reducing stasis, detumescence and as an analgesic agent in China for a long time [5]. Pan *et al.* reported that laetispicine was effective in producing antidepressant and antinociceptive effects and hypothesized that laetispicine was possibly acting on the monoaminergic neurotransmission system to mediate the antidepressant and antinociceptive disorders [6].

The structure of laetispicine is unique. Unlike other antidepressants, laetispicine does not contain an acyclic or aromatic amine. Moreover, it contains an unconjugated allylbenzene motif that has little precedent in natural products. However, molecules containing 1,3-benzodioxole have been shown to induce oxidative damage *in vivo* [7]. To better understand the SAR of laetispicine and explore the potential of laetispicine derivatives as new potent and safe antidepressants, we developed a synthetic approach that would facilitate the synthesis of laetispicine analogues. By using this strategy, laetispicine and eight of its derivatives were synthesized efficiently. Here, we first report the synthesis of laetispicine and several derivatives and their preliminary antidepressant activities evaluated in a forced swimming test.

## 2. Results and Discussion

**Scheme 1:**
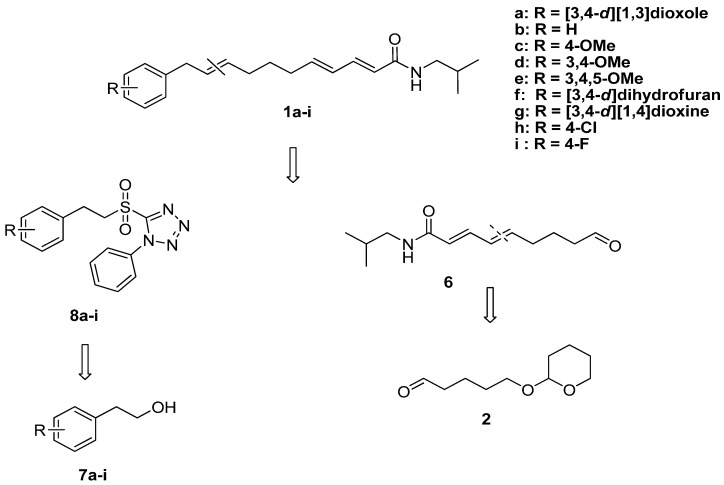
Retro-synthetic analysis of laetispicine and its derivatives.

As shown in Scheme 1, our approach to the synthesis of laetispicine and its derivatives involves the modified Julia-Kocienski olefination as the key step. By varying the combination of different fragments **8a**–**i**, laetispicine and eight of its analogues were obtained. Our SAR study was mainly focused on replacing the 1,3-benzodioxole ring with halogen and alkoxy groups.

**Scheme 2:**
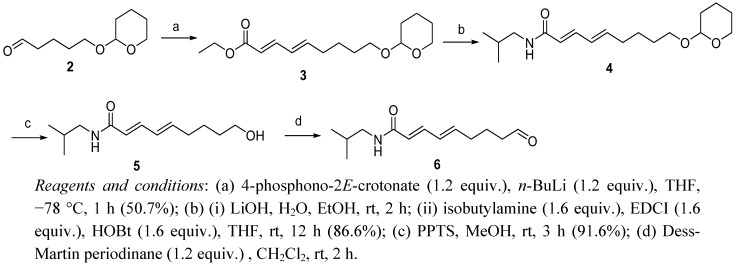
Synthesis of compound **6**.

The synthesis of the key intermediate **6** started from compound **2** [8,9] which reacted with the Hornor-Wadsworth-Emmons reagent to afford the 2*E*,4*E*-diene **3**. No double-bond isomerization was observed [10,11]. Hydrolysis of ester **3** with 1N LiOH gave the corresponding acid, which was condensed with isobutylamine using 1-ethyl-3-(3-dimethylaminopropyl)carbodiimide (EDCI) and hydroxybenzotriazole (HOBt) to give amide **4. **The protecting group of **4** was removed by pyridinium *p*-toluenesulfonate (PPTS) to give **5**, and oxidation of **5** using Dess-Martin periodinane led to the key intermediate aldehyde **6** (Scheme 2).

**Scheme 3:**
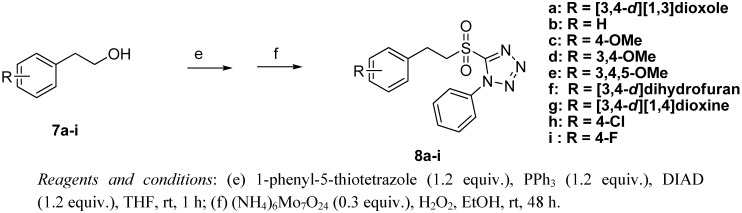
Synthesis of compounds **8a**–**i**.

As shown in Scheme 3, Mitsunobu reaction of commercially available **7a**–**i** with 1-phenyl-5-thiotetrazole by treating with diisopropyl azodicarboxylate (DIAD) afforded the corresponding thioethers, which were then oxidized with hexaammonium heptamolybdate in H_2_O_2 _to give the key intermediates **8a**–**i** [12,13].

**Scheme 4:**
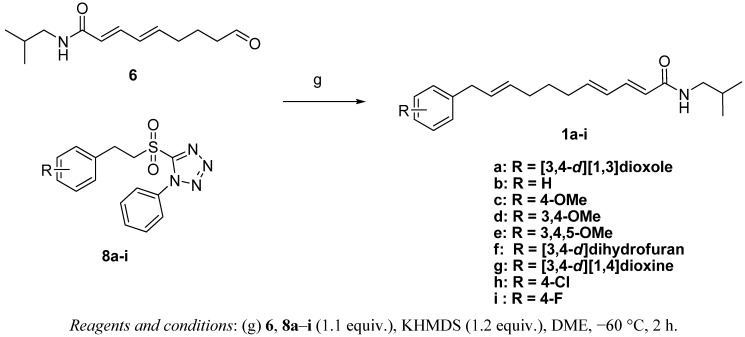
Synthesis of compounds **1a**–**i**.

Compared with the Wittig reaction and Julia-Lythgeo reaction, the modified Julia-Kocienski olefination can afford much higher stereoselectivity in the formation of *E* unconjugated couples [12,13]. Comparing different bases [lithium diisopropylamide (LDA), potassium bis(trimethylsilyl)amide (KHMDS), lithium bis(trimethylsilyl)amide (LHMDS)] and solvents (THF, DME), we found that the reaction of **8a** with **6** by treating with KHMDS in DME furnished laetispicine (**1a**) in good yield (58.2%). Overall, the synthesis of the laetispicine (**1a**) was accomplished in seven steps and 22.1% overall yield with high *E/Z* stereoselectivity (HPLC indicated that *E*:*Z *ratio > 14:1). Synthetic laetispicine was identical in all respects (IR, ^1^H-NMR, ^13^C-NMR, EI, HRMS) with natural laetispicine isolated from *Piper laetispicum* C.DC. [4]. Eight laetispicine derivatives **1b**–**i** were obtained in the same manner (Scheme 4).

Laetispicine (**1a**) and its analogues **1b**–**i** were evaluated for their antidepressant activities in forced swimming test in mice [6,14]. The immobility time of forced swimming mice exposed to **1b**–**i** (10 mg/Kg each) are shown in Table 1. Among the eight derivatives tested, the compounds containing alkoxybenzene moieties showed less antidepressant activities than laetispicine (**1e**, **1g ***vs.***1a**, Table 1), or even lost their antidepressant activities entirely (**1c**, **1d**, **1f*** vs. ***1a**, Table 1). Compound **1b** with no substituent showed similar antidepressant activities as laetispicine. These results suggested that the 1,3-benzodioxole moiety was replaceable, and it was interesting to note that by introducing the halogen atom in the phenyl ring better antidepressant activities than that of laetispicine were achieved (**1h**, **1i*** vs. ***1a**, Table 1). Furthermore, these two compounds with potent antidepressant activities were tested in patch clamp assay to measure their potential to block hERG potassium channel (Table 2). The results showed that **1h** and **1i** had weak inhibition on hERG current (IC_50_ > 100 μM) when compared to the positive reference compound cisapride.

**Table 1:** Effects of compound **1b**–**i **on the forced swimming test in mice (means ± SEM of eight animals). 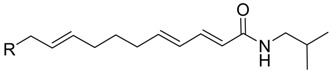

Compound	R	Immobility Time (s)	Reduction (%)
Control		156.0 ± 6.1	
Fluoxetine		161.3 ± 8.1	0
Laetispicine ( **1a**)	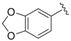	105.2 ± 8.1	33
**1b**	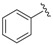	95.6 ± 7.1 ***	39
**1c**	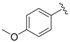	149.2 ± 11.9	5
**1d**	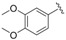	146.1 ± 10.7	6
**1e**	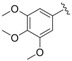	113.4 ± 10.4 **	27
**1f**	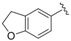	147.5 ± 11.4	5
**1g**	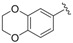	111.2 ± 12.6 **	29
**1h**	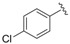	74.6 ± 15.6 ***	52
**1i**	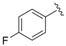	88.5 ± 18.5 ***	43

* *p* < 0.05; ** *p* < 0.01; *** *p* < 0.001 *vs.* control; N = 8 ; AVONA followed by LSD.

**Table 2:** hERG channel binding assay.

Compound	Inhibition % at 1 μM	Inhibition % at 10 μM	Inhibition % at 100 μM
Cisapride	97.9 ± 0.5%		
**1h**	8.0 ± 2.3%	13.9 ± 2.0%	24.2 ± 4.9%
**1i**	7.1 ± 0.7%	10.7 ± 0.1%	17.7 ± 1.5%

## 3. Experimental

### 3.1. General

IR spectra were recorded on a Nicolet Magna-FTIR-750 spectrophotometer. ^1^H- and ^13^C-NMR spectra were recorded in CDCl_3_ on Varian Mercury-300 or Varian Mercury-400 instruments. The ESI-MS were carried out on Thermo Finnigan LCQDECAXP and the low-resolution EI-MS was measured on a MAT-95 spectrometer and HREI-MS on a MAT-77 spectrometer. Purity was recorded on Gilson high-performance liquid chromatography (HPLC) (306 pump, UV/Vis-156 Detector, 215 liquid handle). TLC was carried out with glass pre-coated silica gel GF_254_ plates. Spots were visualized under UV light. All the solvents and reagents were used directly as obtained commercially unless otherwise noted.

*Ethyl 9-(tetrahydropyran-2-yloxy)-2*E*,4*E*-nonadienoate* (**3**). To a solution of triethyl 4-phosphono-2*E*-crotonate (4.3 mL, 19.4 mmol) in anhydrous THF (50 mL) at −78 °C under Ar was added *n*-BuLi (12.1 mL of a 1.6 M solution in hexane, 19.4 mmol). The reaction mixture was stirred at this temperature for 30 min, and then **2** [8,9] (3.0 g, 16.1 mmol) in anhydrous THF (20 mL) was added at −78 °C. The reaction mixture was stirred for an additional 1 h at this temperature, then stirred for 1 h at room temperature. The reaction was quenched with saturated NH_4_Cl (30 mL). Then the mixture was extracted with CH_2_Cl_2 _(3 × 30 mL). The combined organic phases were washed with brine (2 × 50 mL), dried (Na_2_SO_4_), and evaporated. The residue was flash chromatographed (10:1 petroleum ether-EtOAc) to afford **3** as a colorless liquid (2.3 g, 50.7%): ^1^H-NMR (CDCl_3_) *δ* 1.27 (t, 3H, *J* = 7.1 Hz), 1.48–1.81 (m, 10H), 2.18 (m, 2H), 3.34–3.49 (m, 2H), 3.70–3.84 (m, 2H), 4.17 (m, 2H, *J* = 7.1 Hz), 4.55 (m, 1H), 5.75 (d, 1H, *J* = 15.3 Hz), 6.15 (m, 2H), 7.24 (dd, 1H, *J *= 15.4 Hz, *J* = 14.0 Hz); ^13^C-NMR (CDCl_3_) *δ* 14.2, 19.6, 25.4, 29.2, 30.7, 32.7, 60.1, 62.3, 67.2, 98.8, 119.2, 128.5, 144.2, 144.9, 167.2; IR (KBr) *v_max_* 3117, 2941, 1714, 1643, 1618, 1404, 1259, 1138, 1034, 870 cm^−1^.

N*-Isobutyl-9-(tetrahydropyran-2-yloxy)-2*E*,4*E*-nonadienamide* (**4**). To an ice-cooled solution of **3** (1.0 g, 3.55 mmol) in EtOH (20 mL) was added 1 N LiOH (17.8 mL, 17.8 mmol) and the solution was stirred at room temperature for 12 h. The reaction mixture was diluted with H_2_O (20 mL) and washed with EtOAc (2 × 20 mL). The aqueous layer was acidified (pH 2.0) with 2 N HCl and extracted with EtOAc (3 × 20 mL). The combined organic phases were washed with brine (2 × 50 mL), dried (Na_2_SO_4_), and evaporated to give 9-(tetrahydropyran-2-yloxy)-2*E*,4*E*-nonadienoic acid as a yellow solid. The acid was used directly in the next step. The acid, EDCI (1.1 g, 5.68 mmol), HOBt (0.77 g, 5.68 mmol) and isobutylamine (0.56 mL, 5.68 mmol) were dissolved in anhydrous THF (30 mL), and the reaction mixture was stirred at room temperature for 12 h. The reaction mixture was diluted with H_2_O (30 mL) and extracted with CH_2_Cl_2_ (2 × 30 mL). The combined organic phases were washed with brine (2 × 50 mL), dried (Na_2_SO_4_), and evaporated. The residue was flash chromatographed (5:1 petroleum ether-EtOAc) to afford **4** as a colorless oil (0.95 g, two step total yield 86.6%); ^1^H-NMR (CDCl_3_) *δ* 0.92 (d, 6H, *J* = 6.7 Hz), 1.51–1.84 (m, 11H), 2.18 (m, 2H), 3.16 (t, 2H, *J* = 6.5 Hz), 3.47 (m, 2H), 3.75 (m, 2H), 4.56 (m, 1H), 5.53 (brs, 1H), 5.74 (d, 1H, *J* = 14.9 Hz), 6.09 (m, 2H), 7.19 (dd, 1H, *J* = 14.8 Hz, *J* = 14.8 Hz); ^13^C-NMR (CDCl_3_) *δ *19.6, 20.1, 25.4, 25.4, 28.5, 29.2, 30.7, 32.7, 46.8, 62.3, 67.2, 98.8, 121.9, 128.4, 141.0, 142.5, 166.3; IR (KBr) *v_max_* 3282, 2953, 2870, 1659, 1630, 1551, 1352, 1261, 1121, 1034, 999 cm^−1^; ESIMS *m/z* 332 [M + Na]^+^, 310 [M + H]^+^; HREIMS: calcd for C_18_H_31_NO_3_Na [M + Na]^+^, 332.2202; found, 332.2201.

N*-Isobutyl-9-hydroxy-2*E*,4*E*-nonadienamide* (**5**). To a solution of **4** (0.60 g, 1.94 mmol) in MeOH (20 mL) was added PPTS (0.1 g), and the reaction mixture was stirred at room temperature for 3 h. The solvent was evaporated and then H_2_O (20 mL) and CH_2_Cl_2_ (20 mL) were added. The phases were separated, and the aqueous layer was extracted with CH_2_Cl_2_ (2 × 30 mL). The combined organic phases were washed with brine (50 mL), dried (Na_2_SO_4_), and evaporated to give **5** as a colorless oil (0.40 g, 91.6%); ^1^H-NMR (CDCl_3_) *δ* 0.92 (d, 6H, *J* = 6.6 Hz), 1.47–1.63 (m, 4H), 1.77 (m, 1H), 2.18 (m, 2H), 3.16 (m, 2H), 3.65 (t, 2H, *J* = 6.3 Hz), 5.54 (brs, 1H), 5.74 (d, 1H, *J* = 14.9 Hz), 6.09 (m, 2H), 7.22 (dd, 1H, *J* = 14.8 Hz, *J* = 14.9 Hz); ^13^C-NMR (CDCl_3_) *δ* 20.1, 24.9, 28.5, 32.1, 32.6, 46.9, 62.5, 122.0, 128.5, 141.0, 142.4, 166.5; IR (KBr) *v_max_* 3290, 2931, 2870, 1659, 1630, 1551, 1404, 1265, 1161, 1061, 999 cm^−1^; ESIMS *m/z* 451 [2M + H]^+^, 226 [M + H]^+^; HREIMS: calcd for C_13_H_23_NO_2_Na [M + Na]^+^, 248.1626; found, 248.1624.

N*-Isobutyl-9-oxo-2*E*,4*E*-nonadienamide* (**6**). Compound **5** (0.40 g, 1.78 mmol) in CH_2_Cl_2_ (10 mL) was added to the solution of Dess-Martin periodinane (0.90 g, 2.13 mmol) in CH_2_Cl_2 _(20 mL) at 0 °C, and the mixture was stirred at room temperature for 2 h. Saturated Na_2_S_2_O_3_ aqueous solution (20 mL) and CH_2_Cl_2_ (20 mL) were added. The phases were separated, and the aqueous layer was extracted with CH_2_Cl_2_ (2 × 30 mL). The combined organic phases were washed with brine (50 mL), dried (Na_2_SO_4_), and evaporated to give **6** as a yellow solid. The crude aldehyde was used directly in the next step.

*5-(2-(3,4-Methylenedioxyphenyl)ethylsulfonyl)-1-phenyltetrazole* (**8a**). To an ice-cooled solution of **7a** (2.2 g, 13.3 mmol), 1-phenyl-5-thioltetrazole (2.6 g, 14.6 mmol) and triphenylphosphine (3.8 g, 15 mmol) in anhydrous THF (150 mL) under Ar was added DIAD (2.9 mL, 14.6 mmol) in anhydrous THF (30 mL) over 20 min. The reaction mixture was stirred at room temperature for 1 h and the solvent was evaporated. H_2_O (50 mL) and Et_2_O (50 mL) were added to the residue. The phases were separated and the aqueous layer was extracted with Et_2_O (2 × 30 mL). The combined organic phases were washed with brine (50 mL), dried (Na_2_SO_4_), and evaporated. The residue was flash chromatographed (5:1 petroleum ether-EtOAc) to afford crude 5-(2-(3,4-methylenedioxyphenyl)-ethylthio)-1-phenyltetrazole as a white solid. 

To an ice-cooled solution of the thioether (4.1 g, 12.6 mmol) in EtOH (15 mL) under Ar was added hexaammonium heptamolybdate tetrahydrate (4.7 g, 3.8 mmol) in H_2_O_2_ (5 mL) over 10 min. The reaction mixture was stirred at room temperature for 48 h. H_2_O (50 mL) and Et_2_O (50 mL) were added to the mixture. The phases were separated and the aqueous layer was extracted with Et_2_O (2 × 30 mL). The combined organic phases were washed with brine (50 mL), dried (Na_2_SO_4_), and evaporated. The residue was flash chromatographed (5:1 petroleum ether-EtOAc) to afford **8a** as a white solid (4.1 g, 94.6%); m.p. 95–97 °C; ^1^H-NMR (CDCl_3_) *δ* 3.19 (t, 2H, *J* = 8.1 Hz), 3.96 (t, 2H, *J* = 8.1 Hz), 5.96 (s, 2H), 6.72–6.75 (m, 3H), 7.61–7.71 (m, 5H); ^13^C-NMR (CDCl_3_) *δ* 28.3, 57.4, 101.1, 108.6, 108.8, 121.6, 125.0, 129.7, 129.8, 131.5, 132.9, 146.8, 148.0, 153.3; IR (KBr) *v_max_* 2926, 1593, 1504, 1450, 1354, 1248, 1148, 1038, 918, 766, 642, 523 cm^−1^; EIMS *m/z* 358 [M]^+^, 148 (100%); HREIMS: calcd for C_16_H_14_N_4_O_4_S, 358.0736; found, 358.0742.

*Laetispicine* (**1a**). To a solution of **8a** (0.87 g, 2.44 mmol) in anhydrous DME (30 mL) at −60 °C under Ar was added KHMDS (2.7 mL of a 1 M solution in hexane, 2.70 mmol). The reaction mixture was stirred at this temperature for 1 h, and then **6** (0.50 g, 2.22 mmol) in anhydrous DME (10 mL) was added at −60 °C. The reaction mixture was stirred for an additional 2 h at this temperature, then stirred for 30 min at room temperature. The reaction was quenched with saturated NH_4_Cl (30 mL). Then the mixture was extracted with Et_2_O (3 × 30 mL). The combined organic phases were washed with brine (2 × 50 mL), dried (Na_2_SO_4_), and evaporated. The residue was flash chromatographed (3:1 petroleum ether-EtOAc) to afford **1a** as a white solid (0.46 g, two step total yield 58.2%); m.p. 95–96 °C (lit. [4] 93–94 °C); ^1^H-NMR (400 MHz, CDCl_3_) *δ* 0.92 (d, 6H, *J* = 6.6 Hz), 1.50 (m, 2H), 1.81 (m, 1H), 2.03 (m, 2H), 2.15 (m, 2H), 3.15 (t, 2H, *J* = 6.3 Hz), 3.23 (d, 2H, *J* = 5.7 Hz), 5.52 (m, 2H, *J* = 15.0 Hz, *J* = 15.4 Hz), 5.74 (d, 1H, *J* = 14.7 Hz), 5.91 (s, 2H), 6.06 (m, 2H, *J* = 15.0 Hz, *J* = 15.0 Hz), 6.60–6.74 (m, 3H), 7.17 (dd, 1H, *J* = 15.0 Hz, *J* = 9.9 Hz); ^13^C-NMR (CDCl_3_) *δ* 20.1, 28.4, 28.6, 31.8, 32.3, 38.7, 46.9, 100.7, 108.1, 108.9, 121.1, 121.9, 128.5, 129.6, 131.1, 134.7, 141.1, 142.5, 145.6, 147.5, 166.3; IR (KBr) *v_max_* 3425, 3302, 2955, 2922, 1655, 1628, 1614, 1551, 1506, 1487, 1250, 1001, 968 cm^−1^; EIMS *m/z* 355 [M]^+^, 220 (100%); HREIMS: calcd for C_22_H_29_NO_3_, 355.2147; found, 355.2155.

*5-(Phenethylsulfonyl)-1-phenyltetrazole* (**8b**). Mitsunobu reaction of **7b** (5.0 g, 0.041 mol) and then oxidation under similar conditions to those applied for **8a** to afford **8b** as a white solid (8.8 g, 68.3%): m.p. 90–92 °C; ^1^H-NMR (300 MHz, CDCl_3_): *δ* 3.27 (m, 2H), 4.01 (m, 2H), 7.25–7.34 (m, 5H), 7.61–7.72 (m, 5H); ESIMS *m/z* 315 [M + H]^+^.

*5-(4-Methoxyphenethylsulfonyl)-1-phenyltetrazole* (**8c**). Mitsunobu reaction of **7c** (3.9 g, 0.026 mol) and then oxidation under similar conditions to those applied for **8a** to afford **8c** as a white solid (5.9 g, 67.3%); m.p. 99–101 °C; ^1^H-NMR (300 MHz, CDCl_3_) *δ* 3.21 (m, 2H), 3.80 (s, 3H), 3.97 (m, 2H), 6.86 (d, 2H, *J* = 8.4 Hz), 7.17 (d, 2H, *J* = 8.4 Hz), 7.61–7.69 (m, 5H); EIMS *m/z* 344 [M]^+^, 134 (100%).

*5-(3,4-Dimethoxyphenethylsulfonyl)-1-phenyltetrazole* (**8d**). Mitsunobu reaction of **7d** (4.5 g, 0.025 mol) and then oxidation under similar conditions to those applied for **8a** to afford **8d** as a white solid (6.9 g, 75.1%); m.p. 155–157 °C; ^1^H-NMR (300 MHz, CDCl_3_) *δ* 3.21 (m, 2H), 3.87 (s, 3H), 3.88 (s, 3H), 3.98 (m, 2H), 6.75–6.81 (m, 3H), 7.60–7.71 (m, 5H); ESIMS *m/z* 375 [M + H]^+^.

*5-(3,4,5-Trimethoxyphenethylsulfonyl)-1-phenyltetrazole* (**8e**). Mitsunobu reaction of **7e** (4.1 g, 0.019 mol) and then oxidation under similar conditions to those applied for **8a** to afford **8e** as a white solid (5.7 g, 72.6%); m.p. 133–135 °C; ^1^H-NMR (300 MHz, CDCl_3_) *δ* 3.21 (m, 2H), 3.83 (s, 3H), 3.86 (s, 6H), 4.00 (m, 2H), 6.45 (s, 2H), 7.61–7.71 (m, 5H); EIMS *m/z* 404 [M]^+^, 194 (100%).

*5-(2-(2,3-Dihydrobenzofuran-5-yl)ethylsulfonyl)-1-phenyltetrazole* (**8f**). Mitsunobu reaction of **7f** (4.6 g, 0.028 mol) and then oxidation under similar conditions to those applied for **8a** to afford **8f** as a white solid (7.1 g, 71.5%); m.p. 114–115 °C; ^1^H-NMR (300 MHz, CDCl_3_) *δ* 3.17–3.22 (m, 4H), 3.95 (m, 2H), 4.57 (t, 2H, *J* = 6.6 Hz), 6.72 (d, 1H, *J* = 6.0 Hz), 6.97 (m, 1H), 7.09 (s, 1H), 7.58–7.70 (m, 5H); EIMS *m/z* 356 [M]^+^, 146 (100%).

*5-(2-(2,3-Dihydrobenzo* [1,4] *dioxin-6-yl)ethylsulfonyl)-1-phenyltetrazole* (**8g**). Mitsunobu reaction of **7g** (4.0 g, 0.022 mol) and then oxidation under similar conditions to those applied for **8a** to afford **8g** as a white solid (6.0 g, 72.3%); m.p. 113–115 °C; ^1^H-NMR (300 MHz, CDCl_3_) *δ* 3.15 (m, 2H), 3.95 (m, 2H), 4.24 (s, 4H), 6.69–6.83 (m, 3H), 7.60–7.71 (m, 5H); EIMS *m/z* 372 [M]^+^, 162 (100%).

*5-(4-Chlorophenethylsulfonyl)-1-phenyltetrazole* (**8h**). Mitsunobu reaction of **7h** (4.5 g, 0.029 mol) and then oxidation under similar conditions to those applied for **8a** to afford **8h** as a white solid (7.5 g, 74.5%): m.p. 95–97 °C; ^1^H-NMR (300 MHz, CDCl_3_) *δ* 3.26 (m, 2H), 3.98 (m, 2H), 7.19–7.33 (m, 4H), 7.61–7.71 (m, 5H); EIMS *m/z* 349 [M]^+^, 138 (100%).

*5-(4-Fluorophenethylsulfonyl)-1-phenyltetrazole* (**8i**). Mitsunobu reaction of **7i** (4.7 g, 0.034 mol) and then oxidation under similar conditions to those applied for **8a** to afford **8i** as a white solid (7.6 g, 67.3%); m.p. 99–100 °C; ^1^H-NMR (400 MHz, CDCl_3_) *δ* 3.25 (m, 2H), 3.99 (m, 2H), 7.02 (m, 2H), 7.24 (m, 2H), 7.59–7.70 (m, 5H); ESIMS *m/z* 333 [M + H]^+^.

N*-Isobutyl-11-phenylundeca-2*E*,4*E*,9*E*-trienamide* (**1b**). Julia reaction of **8b** (0.15 g, 0.49 mmol) under similar conditions to those applied for latispicine (**1a**) afforded **1b** as a white solid (72 mg, 51.2%); m.p. 80–82 °C; ^1^H-NMR (300 MHz, CDCl_3_) *δ* 0.92 (d, 6H, *J* = 6.6 Hz), 1.51 (m, 2H), 1.80 (m, 1H), 2.03 (m, 2H), 2.16 (m, 2H), 3.16 (t, 2H, *J *= 6.3 Hz), 3.33 (d, 2H, *J* = 6.0 Hz), 5.52 (m, 2H), 5.72 (d, 1H, *J* = 15.0 Hz), 6.08 (m, 2H), 7.14–7.31 (m, 6H); ^13^C-NMR (CDCl_3_) *δ* 20.1, 28.5, 28.6, 31.9, 32.4, 39.1, 47.0, 121.8, 125.9, 128.4, 128.5, 128.5, 129.5, 131.2, 140.9, 141.3, 142.7, 166.4; EIMS *m/z* 311 [M]^+^, 91 (100%); HREIMS: calcd for C_2__1_H_29_NO, 311.2249; found, 311.2258.

N*-Isobutyl-11-(4-methoxyphenyl)undeca-2*E*,4*E*,9*E*-trienamide* (**1c**). Julia reaction of **8c** (0.20 g, 0.58 mmol) under similar conditions to those applied for latispicine (**1a**) afforded **1c **as a white solid (90 mg, 50.1%); m.p. 65–67 °C; ^1^H-NMR (300 MHz, CDCl_3_) *δ* 0.92 (d, 6H, *J* = 6.6 Hz), 1.50 (m, 2H), 1.80 (m, 1H), 2.01 (m, 2H), 2.15 (m, 2H), 3.16 (t, 2H, *J* = 6.6 Hz), 3.26 (d, 2H, *J* = 6.3 Hz), 3.79 (s, 3H), 5.45–5.54 (m, 2H), 5.72 (d, 1H, *J* = 15.0 Hz), 6.04–6.10 (m, 2H), 6.83 (d, 2H, *J* = 8.7 Hz), 7.08 (d, 2H, *J* = 8.4 Hz), 7.18 (m, 1H); ^13^C-NMR (CDCl_3_) *δ* 20.2, 28.5, 28.6, 31.9, 32.3, 38.1, 46.9, 55.3, 113.8, 122.0, 128.5, 129.4, 129.9, 130.8, 133.0, 141.1, 142.6, 157.8, 166.4; EIMS *m/z* 341 [M]^+^, 121 (100%); HREIMS: calcd for C_22_H_31_NO_2_, 341.2355; found, 341.2360.

N*-Isobutyl-11-(3,4-dimethoxyphenyl)undeca-2*E*,4*E*,9*E*-trienamide* (**1d**). Julia reaction of **8d** (0.19 g, 0.51 mmol) under similar conditions to those applied for latispicine (**1a**) afforded **1d** as a white solid (85 mg, 49.4%); m.p. 61–63 °C; ^1^H-NMR (300 MHz, CDCl_3_) *δ* 0.92 (d, 6H, *J* = 6.9 Hz), 1.50 (m, 2H), 1.78 (m, 1H), 2.03 (m, 2H), 2.16 (m, 2H), 3.16 (t, 2H, *J* = 6.3 Hz), 3.26 (d, 2H, *J* = 6.3 Hz), 3.86 (s, 3H), 3.86 (s, 3H), 5.47–5.53 (m, 2H), 5.70 (d, 1H, *J* = 14.7 Hz), 6.06–6.09 (m, 2H), 6.70–6.81 (m, 3H), 7.18 (m, 1H); ^13^C-NMR (100 MHz, CDCl_3_) *δ* 20.1, 28.5, 28.6, 31.9, 32.3, 38.6, 46.9, 55.8, 56.0, 111.2, 111.8, 120.2, 121.9, 128.6, 129.8, 131.0, 133.6, 141.1, 142.5, 147.2, 148.8, 166.3; EIMS *m/z* 371 [M]^+^, 151 (100%); HREIMS: calcd for C_23_H_33_NO_3_, 371.2460; found, 371.2463.

N*-Isobutyl-11-(3,4,5-trimethoxyphenyl)undeca-2*E*,4*E*,9*E*-trienamide*** (1e)**. Julia reaction of **8e** (0.20 g, 0.50 mmol) under similar conditions to those applied for latispicine (**1a**) afforded **1e** as a white solid (92 mg, 50.5%); m.p. 72–73 °C; ^1^H-NMR (300 MHz, CDCl_3_) *δ* 0.92 (d, 6H, *J* = 6.6 Hz), 1.52 (m, 2H), 1.79 (m, 1H), 2.05 (m, 2H), 2.17 (m, 2H), 3.16 (t, 2H, *J* = 6.6 Hz), 3.26 (d, 2H, *J* = 5.4 Hz), 3.82 (s, 3H), 3.84 (s, 6H), 5.52 (m, 2H), 5.68 (d, 1H, *J* = 14.7 Hz), 6.06 (m, 2H), 6.40 (s, 2H), 7.17 (m, 1H); ^13^C-NMR (CDCl_3_) *δ* 20.1, 28.4, 28.6, 31.8, 32.3, 39.4, 46.9, 56.0, 60.9, 105.3, 122.0, 128.7, 129.5, 131.3, 135.9, 136.8, 141.1, 142.4, 153.1, 166.4; EIMS *m/z* 401 [M]^+^, 181 (100%); HREIMS: calcd for C_24_H_35_NO_4_, 401.2566; found, 401.2567.

N*-Isobutyl-11-(2,3-dihydrobenzofuran-5-yl)undeca-2*E*,4*E*,9*E*-trienamide* (**1f**). Julia reaction of **8f** (0.18 g, 0.51 mmol) under similar conditions to those applied for latispicine (**1a**) afforded **1f **as a white solid (89 mg, 54.4%); m.p. 70–72 °C; ^1^H-NMR (300 MHz, CDCl_3_) *δ* 0.92 (d, 6H, *J* = 6.3 Hz), 1.50 (m, 2H), 1.79 (m, 1H), 2.02 (m, 2H), 2.15 (m, 2H), 3.14–3.25 (m, 6H), 4.54 (t, 2H, *J* = 8.7 Hz), 5.47–5.53 (m, 2H), 5.72 (d, 1H, *J* = 14.7 Hz), 6.08 (m, 2H), 6.70 (d, 1H, *J* = 8.1 Hz), 6.90 (d, 1H, *J* = 8.4 Hz), 7.00 (s, 1H), 7.17 (m, 1H);^ 13^C-NMR (CDCl_3_)*δ* 20.1, 28.5, 28.6, 29.8, 31.9, 32.4, 38.5, 46.9, 71.2, 109.0, 121.9, 125.0, 127.0, 127.8, 128.5, 130.2, 130.7, 132.9, 141.2, 142.7, 158.3, 166.3; EIMS *m/z* 353 [M]^+^, 133 (100%); HREIMS: calcd for C_23_H_31_NO_2_, 353.2355; found, 353.2362.

N*-Isobutyl-11-(2,3-dihydrobenzo* [1,4] *dioxin-6-yl)undeca-2*E*,4*E*,9*E*-trienamide* (**1g**). Julia reaction of **8g** (0.15 g, 0.40 mmol) under similar conditions to those applied for latispicine (**1a**) afforded **1g** as a white solid (75 mg, 55.9%); m.p. 69–72 °C; ^1^H-NMR (300 MHz, CDCl_3_) *δ* 0.92 (d, 6H, *J* = 6.6 Hz), 1.50 (m, 2H), 1.80 (m, 1H), 2.01 (m, 2H), 2.14 (m, 2H), 3.14–3.22 (m, 4H), 4.23 (s, 4H), 5.45–5.52 (m, 2H), 5.72 (d, 1H, *J* = 14.7 Hz), 6.07 (m, 2H), 6.62–6.79 (m, 3H), 7.18 (m, 1H); ^13^C-NMR (CDCl_3_) *δ* 20.1, 28.5, 28.6, 31.9, 32.4, 38.3, 46.9, 64.3, 64.4, 117.0, 117.1, 121.3, 121.8, 128.5, 129.6, 131.0, 134.3, 141.2, 141.7, 142.7, 143.3, 166.4; EIMS *m/z* 369 [M]^+^, 149 (100%); HREIMS: calcd for C_23_H_31_NO_3_, 369.2304; found, 369.2298.

N*-Isobutyl-11-(4-chlorophenyl)undeca-2*E*,4*E*,9*E*-trienamide* (**1h**). Julia reaction of **8h** (0.17 g, 0.49 mmol) under similar conditions to those applied for latispicine (**1a**) afforded **1h** as a white solid (82 mg, 52.8%); m.p. 70–73 °C; ^1^H-NMR (300 MHz, CDCl_3_) *δ* 0.93 (d, 6H, *J* = 6.6 Hz), 1.52 (m, 2H), 1.81 (m, 1H), 2.04 (m, 2H), 2.15 (m, 2H), 3.17 (t, 2H, *J* = 6.6 Hz), 3.30 (d, 2H, *J* = 5.7 Hz), 5.47–5.57 (m, 2H), 5.73 (d, 1H, *J *= 15.3 Hz), 6.08 (m, 2H), 7.10–7.28 (m, 5H); ^13^C-NMR (CDCl_3_) *δ* 20.1, 28.4, 28.6, 31.9, 32.3, 38.3, 46.9, 122.0, 128.4, 128.6, 129.0, 129.8, 131.6, 131.7, 139.4, 141.1, 142.5, 166.4; EIMS *m/z* 345 [M]^+^, 125 (100%); HREIMS: calcd for C_21_H_28_NOCl, 345.1859; found, 345.1856.

N*-isobutyl-11-(4-fluorophenyl)undeca-2*E*,4*E*,9*E*-trienamide*** (1i)**. Julia reaction of **8i** (0.19 g, 0.57 mmol) under similar conditions to those applied for latispicine (**1a**) afforded **1i** as a white solid (98 mg, 57.5%); m.p. 75–77 °C; ^1^H-NMR (300 MHz, CDCl_3_) *δ* 0.92 (d, 6H, *J* = 6.8 Hz), 1.50 (m, 2H), 1.80 (m, 1H), 2.03 (m, 2H), 2.15 (m, 2H), 3.16 (t, 2H, *J* = 6.4 Hz), 3.29 (d, 2H, *J* = 6.4 Hz), 5.46–5.56 (m, 2H), 5.73 (d, 1H, *J *= 14.8 Hz), 6.08 (m, 2H), 6.95 (m, 2H), 7.10–7.26 (m, 3H); ^13^C-NMR (CDCl_3_) *δ* 20.1, 28.5, 28.6, 31.9, 32.4, 38.2, 46.9, 115.0, 115.2, 121.9, 128.5, 129.4, 129.8, 129.8, 131.4, 141.2, 142.6, 166.3; EIMS *m/z* 329 [M]^+^, 109 (100%); HREIMS: calcd for C_21_H_28_NOF, 329.2155; found, 329.2147.

### 3.2. Animals

KM mice (18–22 g) of either sex were obtained from Laboratory Animal Center of the College of Medicine, Fudan University. Animals were housed in standard environmental conditions with free access to food and water. The animals were used only once throughout the study. They were allowed to acclimatize to the laboratory seven days before pharmacological tests. The experiment procedures were conducted in compliance with the National Institutes of Health Guide for Care and Use of the laboratory Animals.

### 3.3. Forced Swimming Test

Tween 80 was purchased from Sinopharm Chemical Reagent Co., Ltd (Shanghai, China), fluoxetine from Shanghai Zhongxi Pharmaceutical Co., Ltd. All other reagents were of analytical grade. All drugs were dissolved in saline with 2% Tween 80. Drugs (10 mg/kg/2 mL) were administered by intragastric (i.g.) route 60 min before the forced swimming tests. The control group received only saline with 2% Tween 80 simultaneously. 

The forced swimming test adopted here is a modification of the method described by Porsoltetal [14]. Briefly, mice were individually forced to swim for 15 min in glass cylinders (height: 20 cm, diameter: 14 cm), containing 10 cm of water at 25 °C, which is a pre-test, and then mice were removed and dried before being returned to cages. Twenty-four hours later, mice were placed in the cylinders again for a 6-min test in the same system depicted above. The duration of immobility was recorded during the last 4 min of the 6-min testing period.

### 3.4. Statistical Analyses

Data obtained were expressed as mean ± SEM and analyzed by analysis of variance (ANOVA) followed by Bonferroni’s test. *p*-values less than 0.05 (*p* < 0.05) were used as the significant level. The percent of inhibition was determined using the following formula:

Inhibition (%) = 100 × [(control − experiment) / control].

### 3.5. hERG Inhibition

A CHO cell line stably expressing hERG potassium channels were voltage clamped using automated QPatch electrophysiology system. Test items were dissolved in DMSO and diluted with external recording buffer. Cells were exposed to test concentration for approximately 5 min or till a steady state block was reached at 20–35 °C. Each cell acted as its own control. Cisapride (1 M) was used as an internal positive control to confirm the sensitivity of the test system to hERG inhibition. The extent of inhibition of channel was expressed as a percentage of the control response (minus the test compound).

## 4. Conclusions

In conclusion, we have synthesized laetispicine (**1a**) and eight of its derivatives. The synthetic route achieved a good yield and high *E/Z* stereoselectivity (7 steps, 22.1% overall yield and *E*:*Z *ratio > 14:1) for laetispicine (**1a**). This methodology may potentially be applicable to the synthesis of other analogues of this family and facilitate further SAR research on laetispicine derivatives. Based on the outcomes of a forced swimming test and hERG channel binding studies, compounds **1h** and **1i** were identified as potential new lead antidepressant compounds. Further studies based on this kind of scaffold are in progress and will be reported in due course.

## Supplementary Materials

Supplementary materials can be accessed at: http://www.mdpi.com/1420-3049/17/2/1425/s1.
