# Supplementary material for: Total Synthesis and Antidepressant Activities of Laetispicine and Its Derivatives
Source: Molecules. 2012 Feb 3;17(2):1425–36. doi: 10.3390/molecules17021425 (PMC6268093; doi:10.3390/molecules17021425)
Supplement: Supplementary file 1 [file molecules-17-01425-s001.pdf]

Supporting Information

Total Synthesis and Antidepressant Activities of Laetispicine and Its Derivatives

Shuyi Yao [1], Hui Xie [2], Li Zhang [1], Tao Meng [1], Yongliang Zhang [1], Xin Wang [1], Lin Chen [1], Shengli Pan [2] and Jingkang Shen [1,*]

[1] State Key Laboratory of Drug Research, Shanghai Institute of Materia Medica, Chinese Academy of Sciences, 555 Zuchongzhi Road, Shanghai 201203, China

[2] School of Pharmacy, Fudan University, 826 Zhangheng Road, Shanghai 201203, China

* To whom correspondence should be addressed; E-Mail: jkshen@mail.shcnc.ac.cn; Tel.: +86-21-5080-6600 (ext. 5407); Fax: +86-21-5080-7088.

Spectral Data

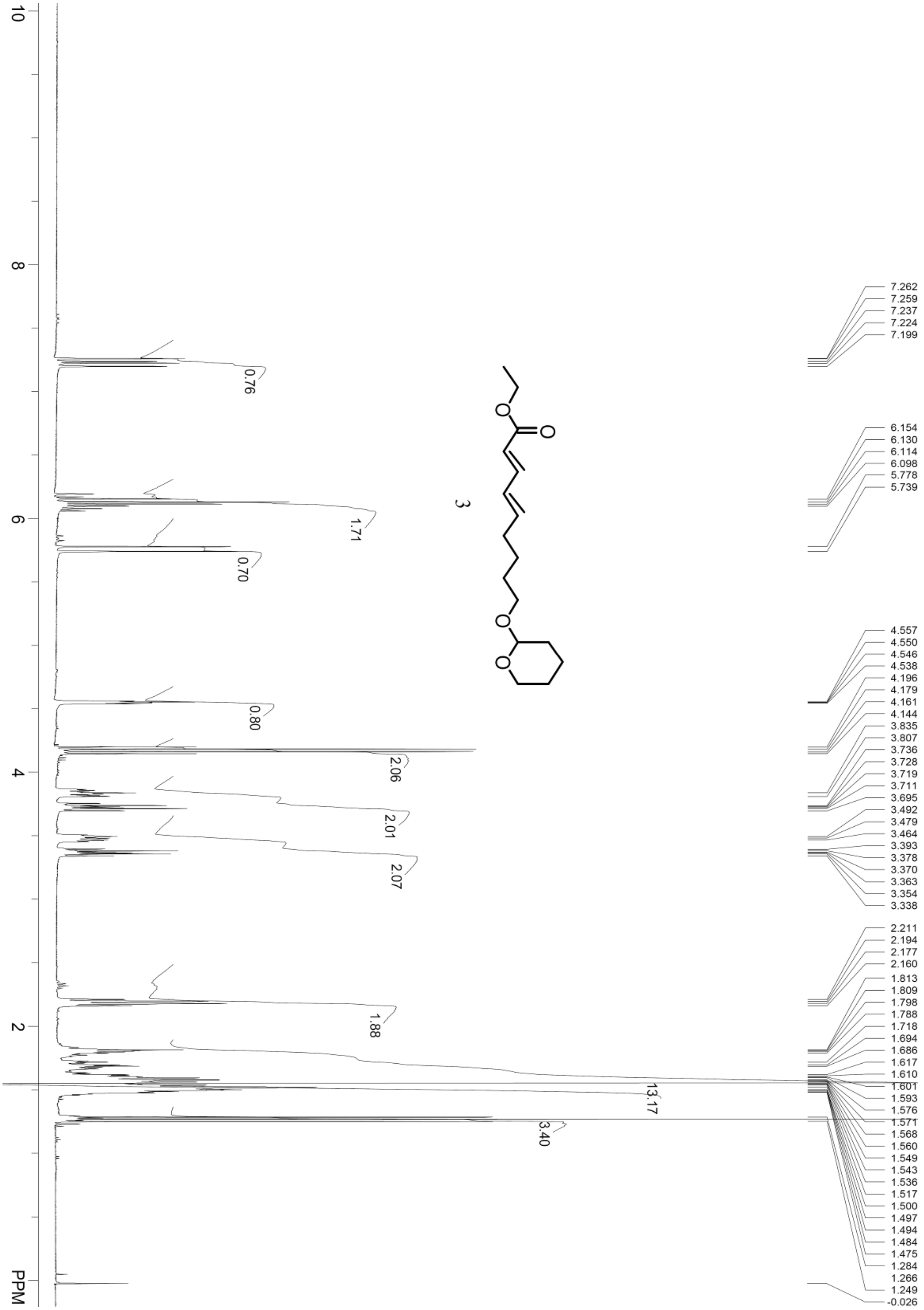

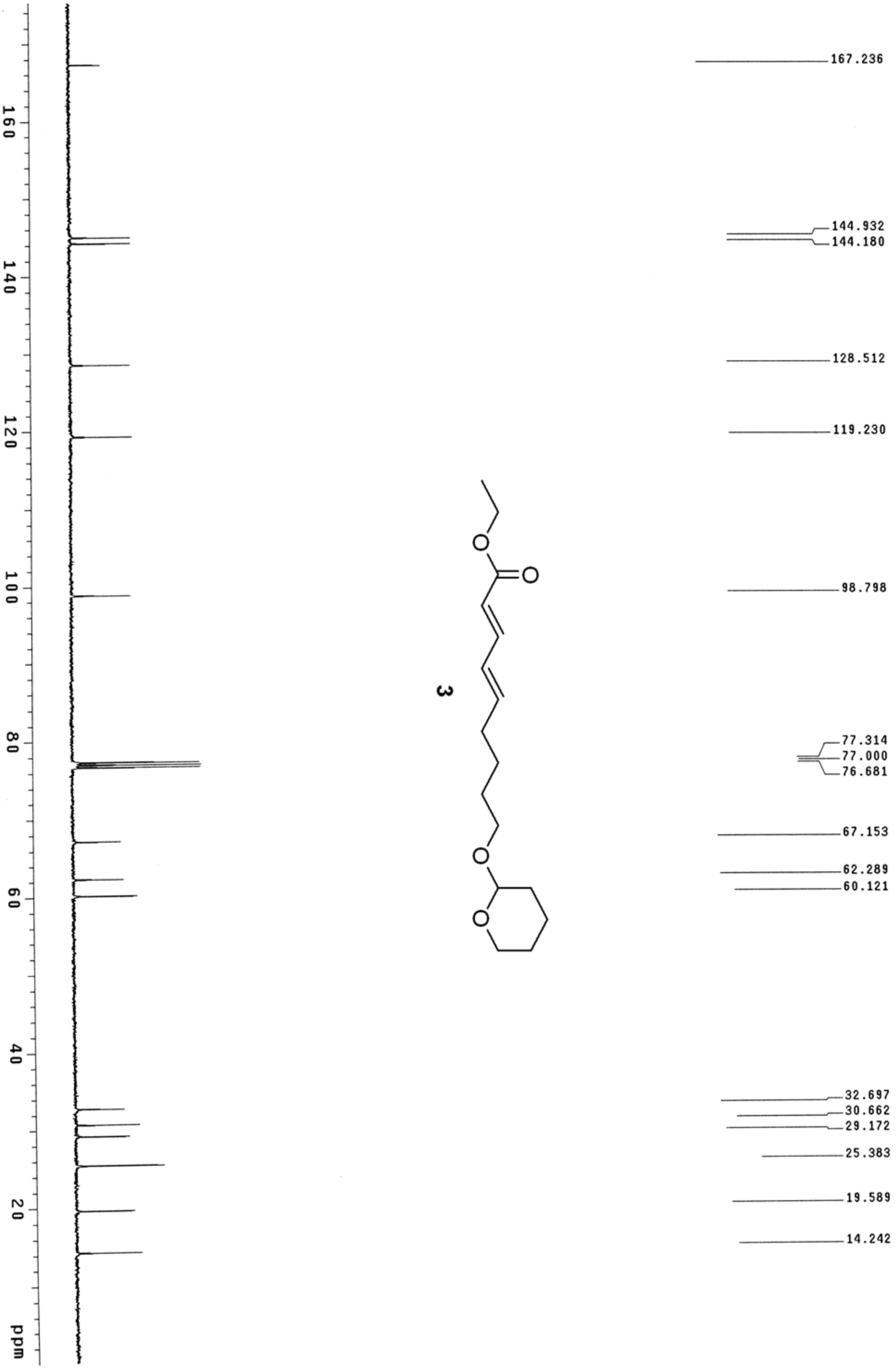
167.236
144.932
144.180
128.512
119.230
98.798
77.314
77.000
76.681
67.153
62.289
60.121
32.697
30.662
29.172
25.383
19.589
14.242
160
140
120
100
80
60
40
20
ppm
3

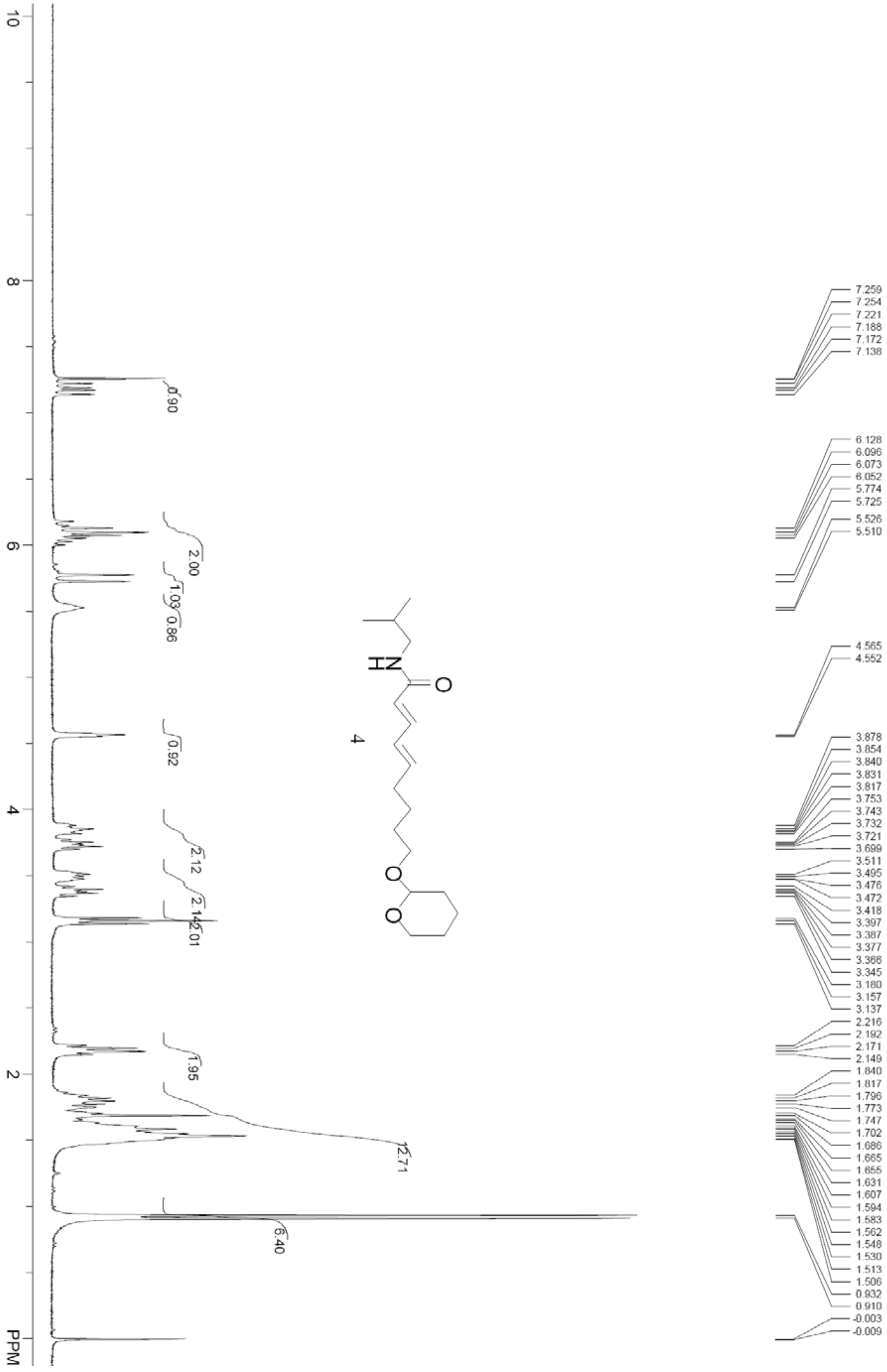
10
8
6
4
2
0
PPM
0.90
2.00
1.03
0.86
0.92
2.12
2.14
2.01
1.95
12.71
6.40
4
7.259
7.254
7.221
7.188
7.172
7.138
6.128
6.096
6.073
6.052
5.774
5.725
5.526
5.510
4.565
4.552
3.878
3.854
3.840
3.831
3.817
3.753
3.743
3.732
3.721
3.699
3.511
3.495
3.476
3.472
3.418
3.397
3.387
3.377
3.366
3.345
3.180
3.157
3.137
2.216
2.192
2.171
2.149
1.840
1.817
1.796
1.773
1.747
1.702
1.686
1.665
1.655
1.631
1.607
1.594
1.583
1.562
1.548
1.530
1.513
1.506
0.932
0.910
-0.003
-0.009

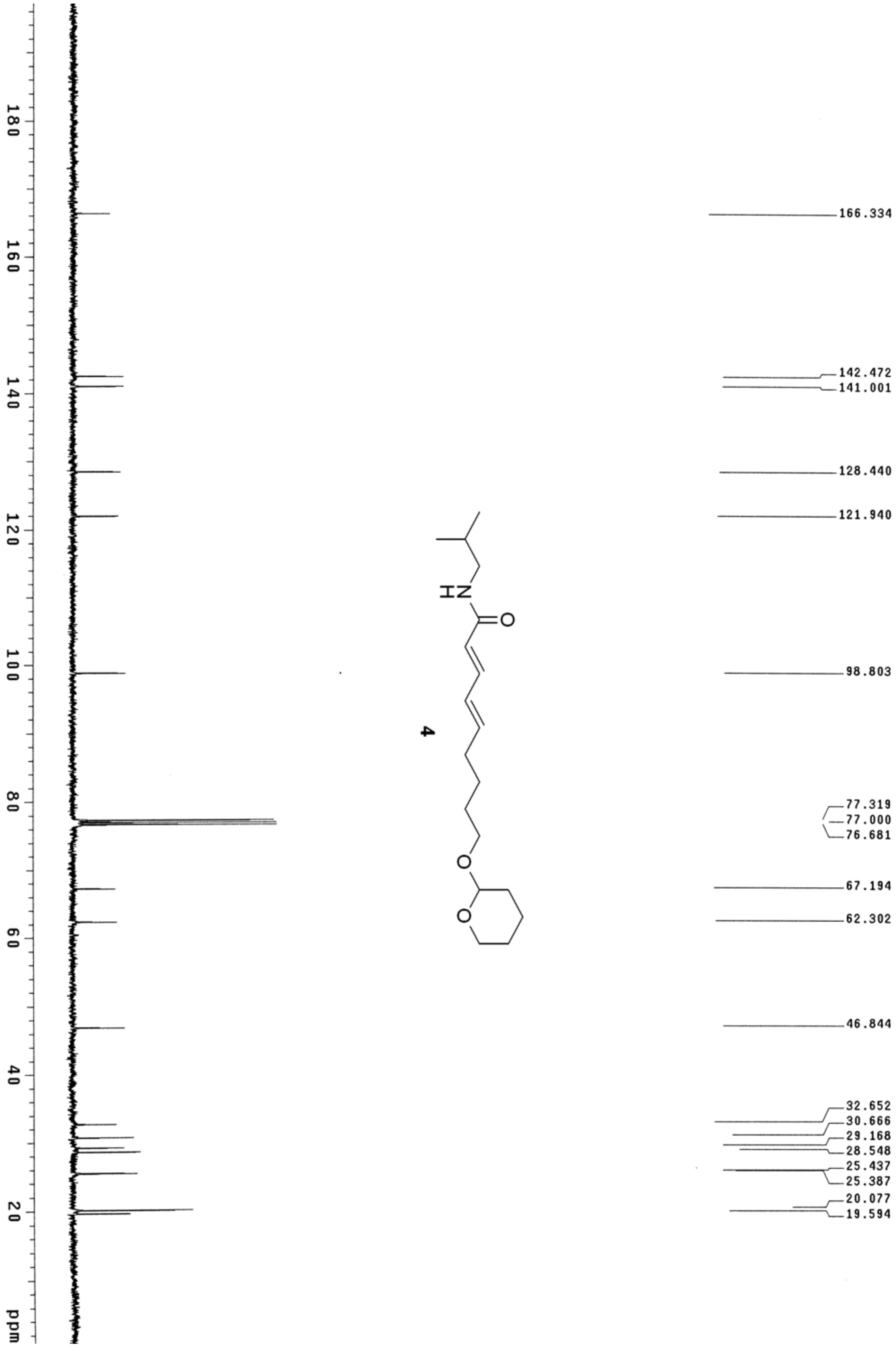
166.334
142.472
141.001
128.440
121.940
98.803
77.319
77.000
76.681
67.194
62.302
46.844
32.652
30.666
29.168
28.548
25.437
25.387
20.077
19.594
4
ppm

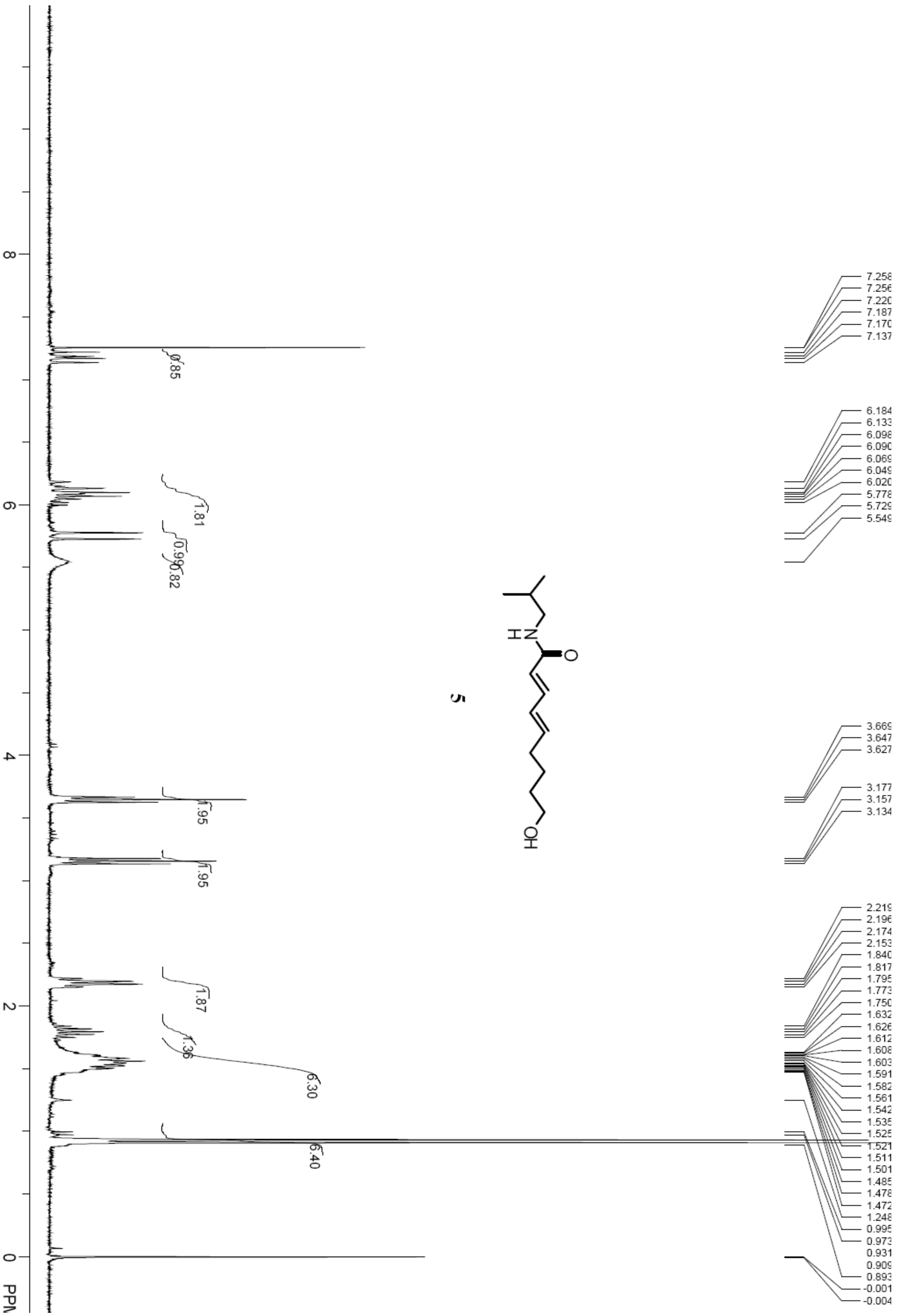
8
6
4
2
0
PPM
0.85
1.81
0.99
0.82
1.95
1.95
1.87
1.36
6.30
6.40
O
N
H
OH
5

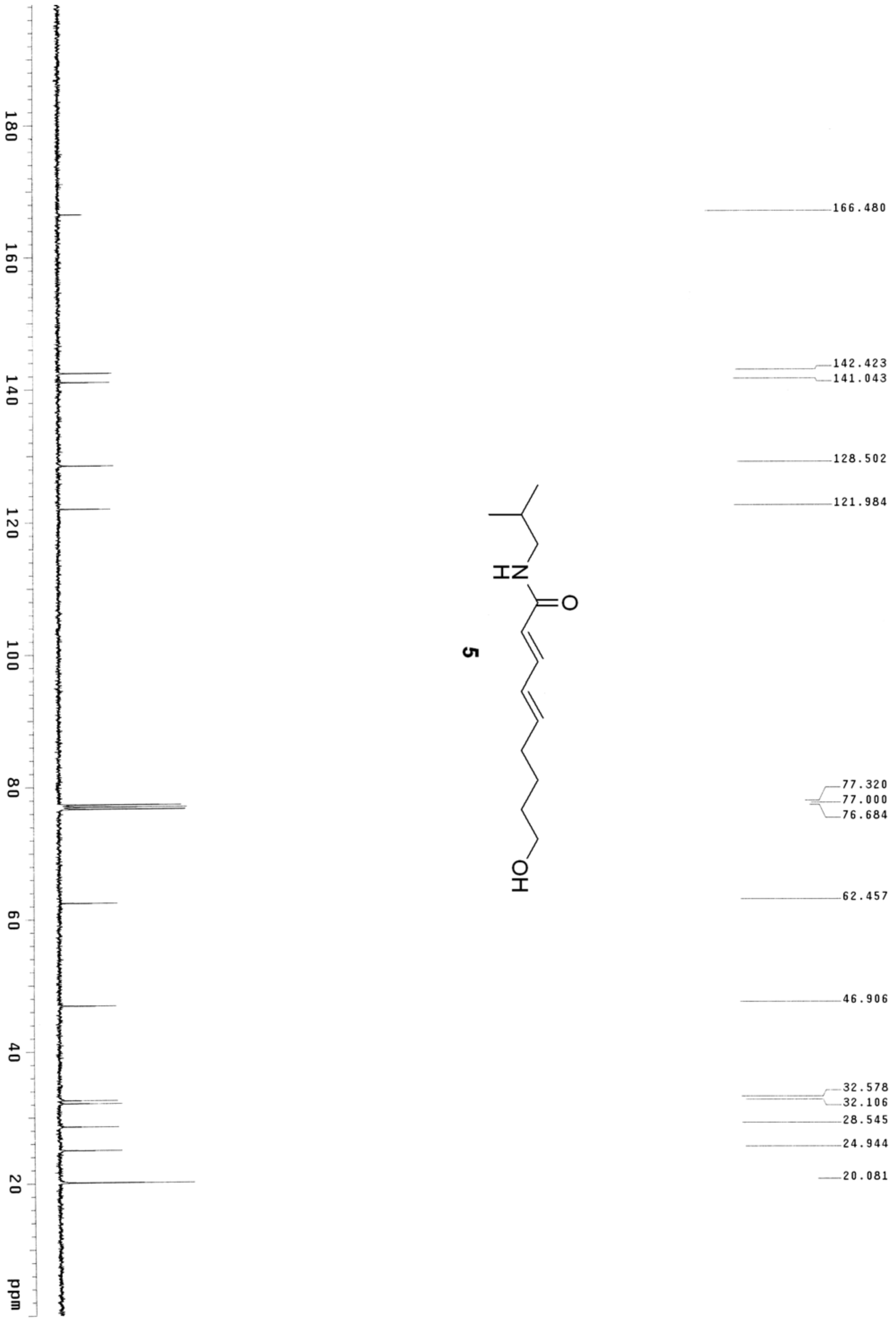
166.480
142.423
141.043
128.502
121.984
77.320
77.000
76.684
62.457
46.906
32.578
32.106
28.545
24.944
20.081
180
160
140
120
100
80
60
40
20
ppm
N
H
O
OH
5

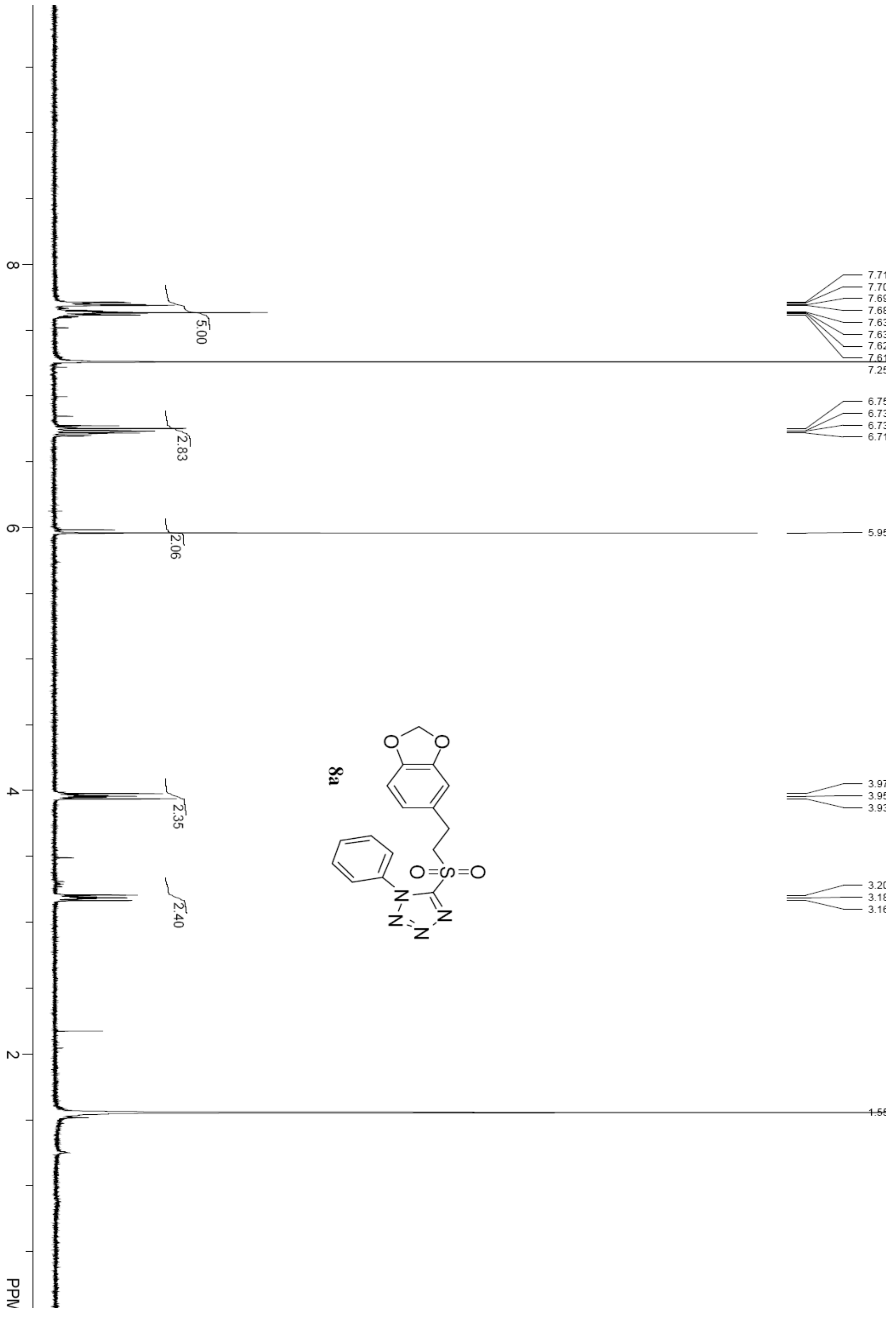
8
6
4
2
PPM
5.00
2.83
2.06
2.35
2.40
8a
7.71
7.25
6.71
1.55

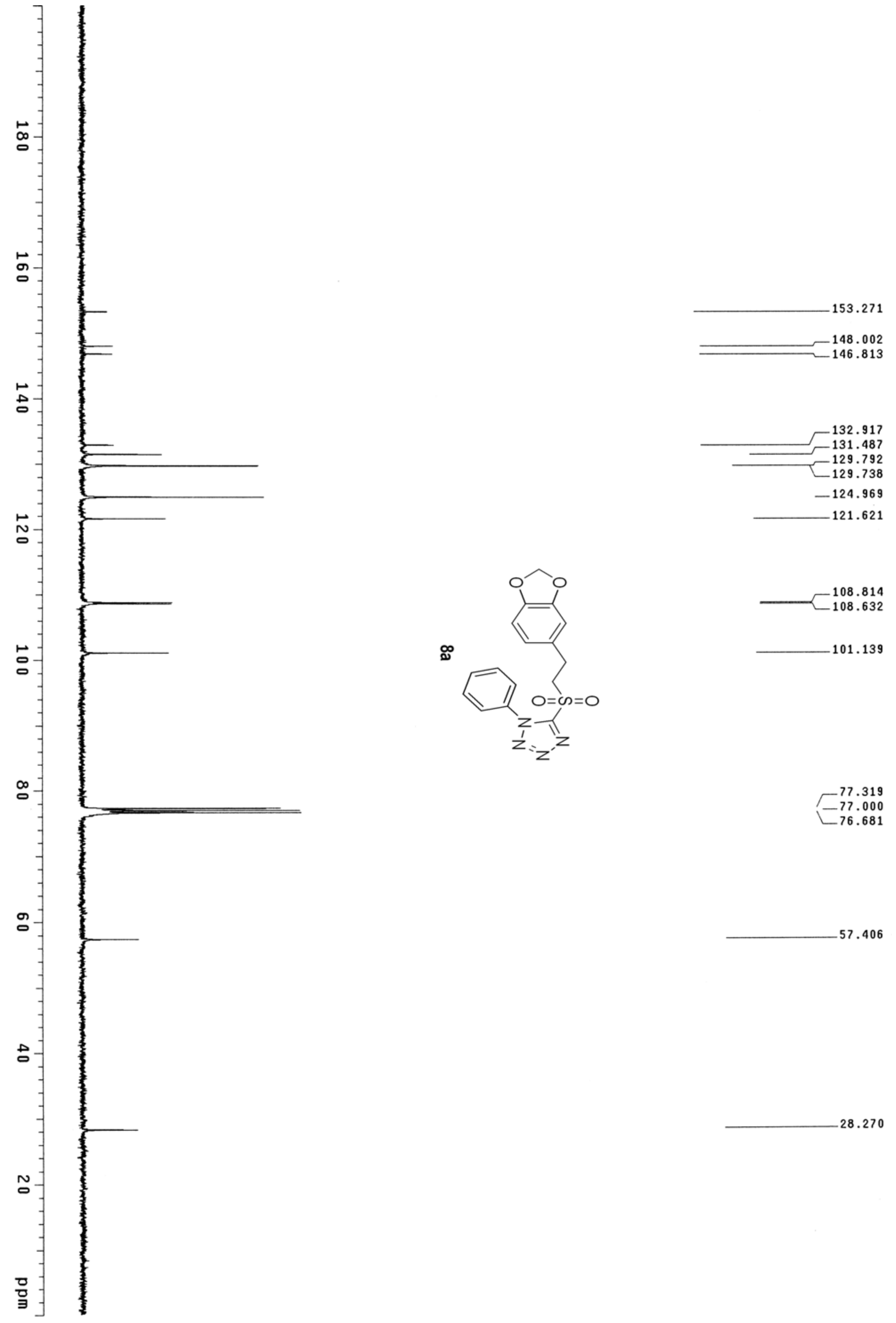
8a
153.271
148.002
146.813
132.917
131.487
129.792
129.738
124.969
121.621
108.814
108.632
101.139
77.319
77.000
76.681
57.406
28.270
180
160
140
120
100
80
60
40
20
ppm

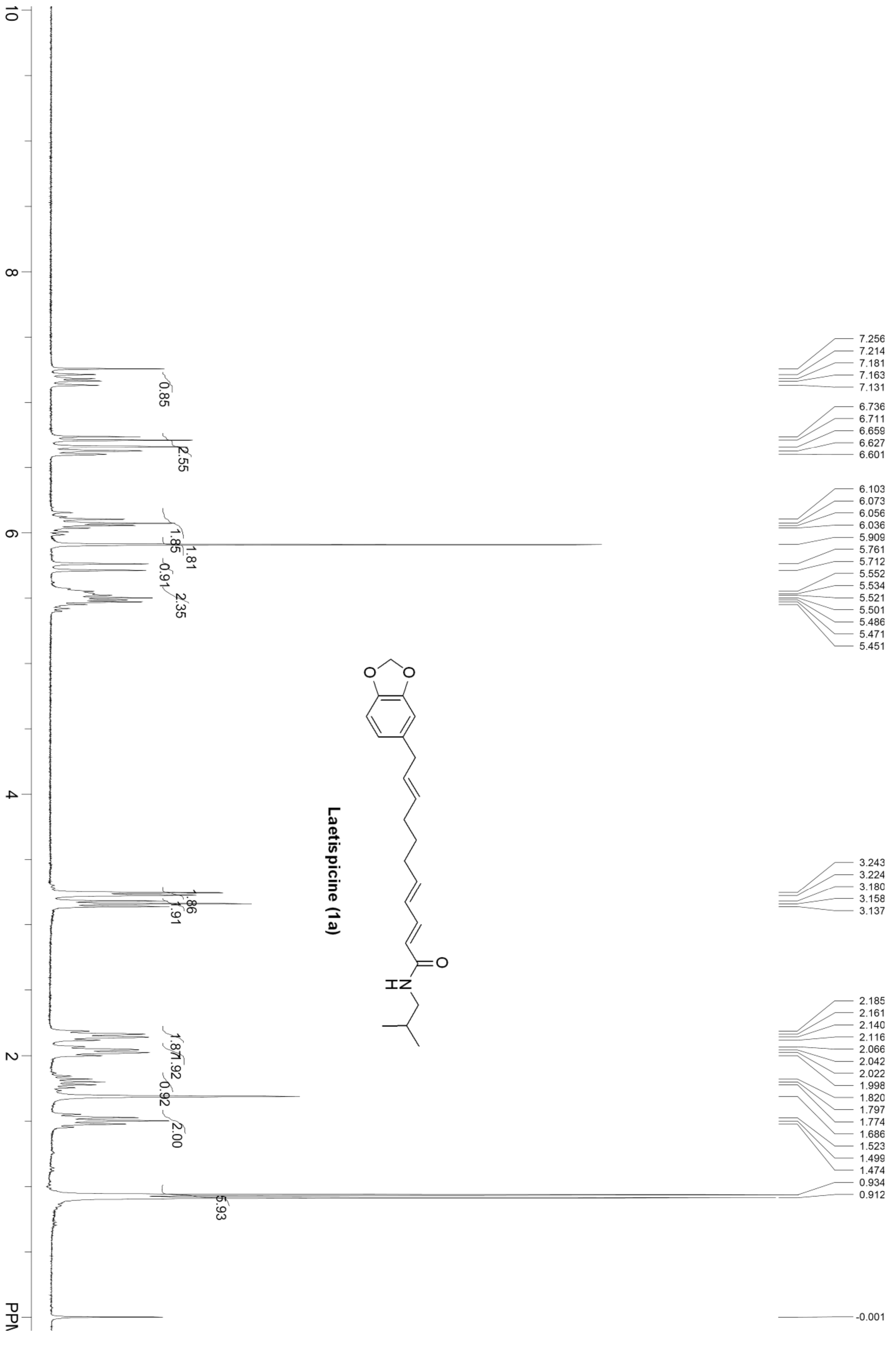
Laetispicine (1a)
PPM
7.256
7.214
7.181
7.163
7.131
6.736
6.711
6.659
6.627
6.601
6.103
6.073
6.056
6.036
5.909
5.761
5.712
5.552
5.534
5.521
5.501
5.486
5.471
5.451
3.243
3.224
3.180
3.158
3.137
2.185
2.161
2.140
2.116
2.066
2.042
2.022
1.998
1.820
1.797
1.774
1.686
1.523
1.499
1.474
0.934
0.912
-0.001

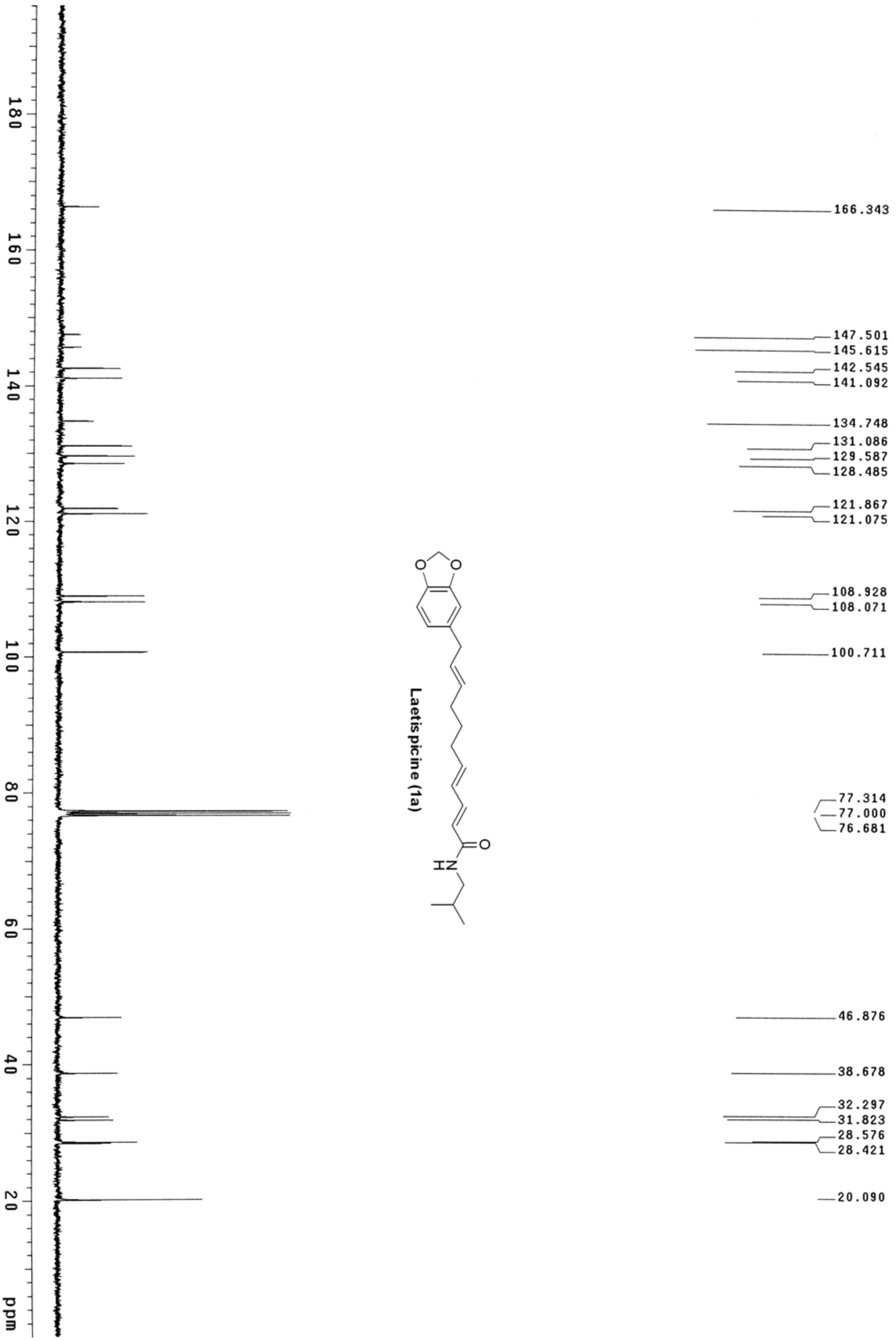
166.343
147.501
145.615
142.545
141.092
134.748
131.086
129.587
128.485
121.867
121.075
108.928
108.071
100.711
77.314
77.000
76.681
46.876
38.678
32.297
31.823
28.576
28.421
20.090
180
160
140
120
100
80
60
40
20
ppm
Laetispicine (1a)

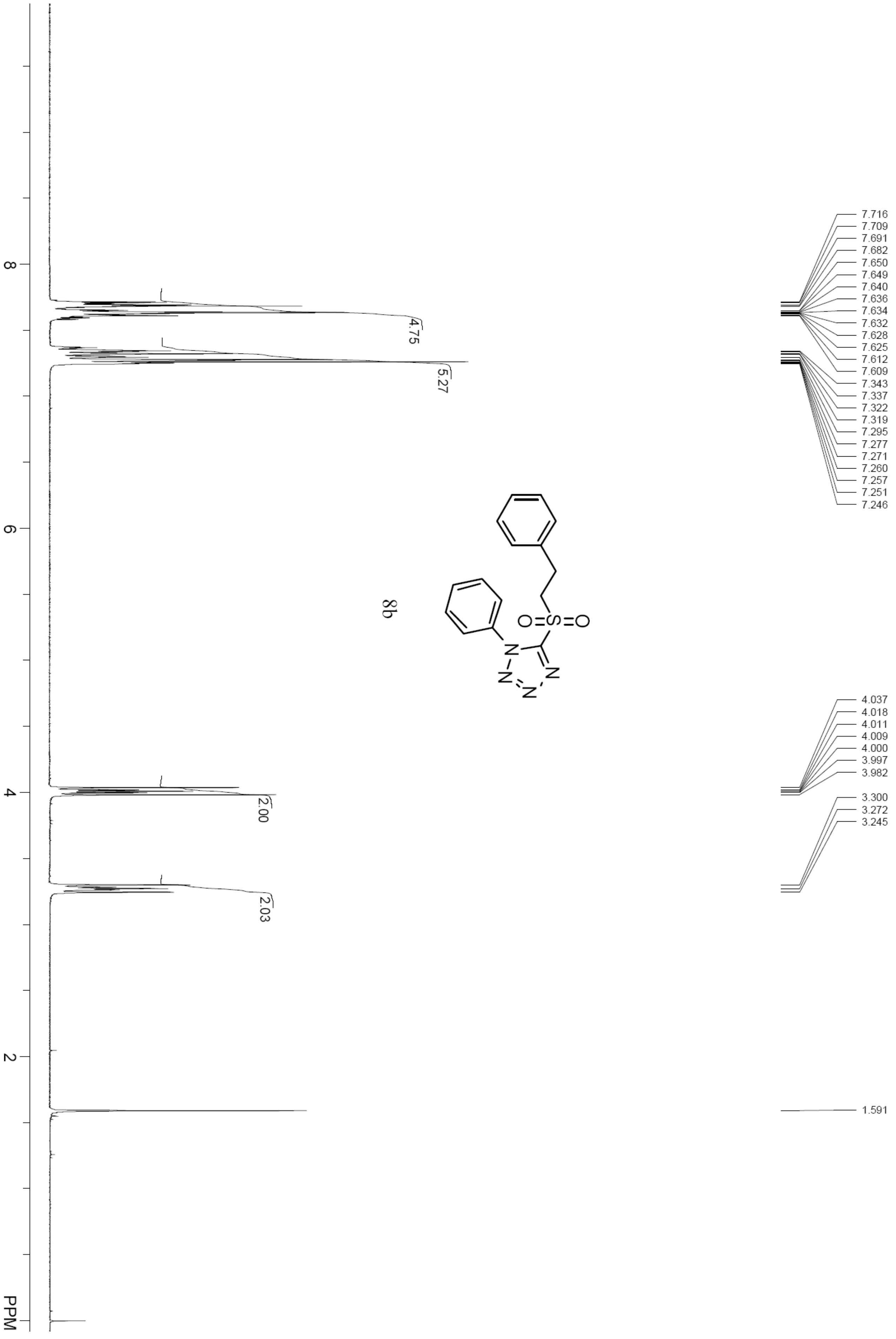
8b
PPM
8
6
4
2
4.75
5.27
2.00
2.03
7.716
7.709
7.691
7.682
7.650
7.649
7.640
7.636
7.634
7.632
7.628
7.625
7.612
7.609
7.343
7.337
7.322
7.319
7.295
7.277
7.271
7.260
7.257
7.251
7.246
4.037
4.018
4.011
4.009
4.000
3.997
3.982
3.300
3.272
3.245
1.591

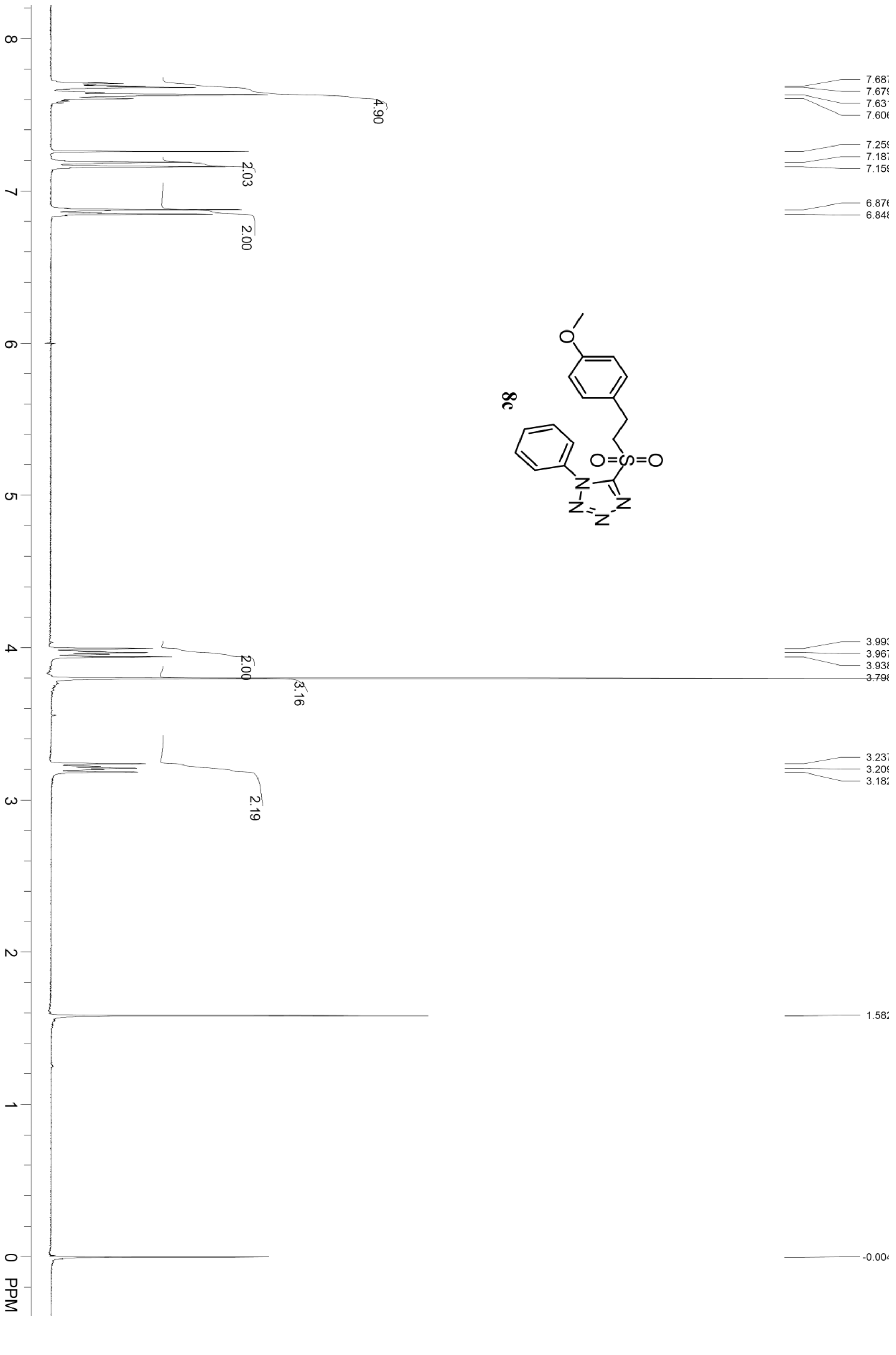
8c
PPM
4.90
2.03
2.00
2.00
3.16
2.19

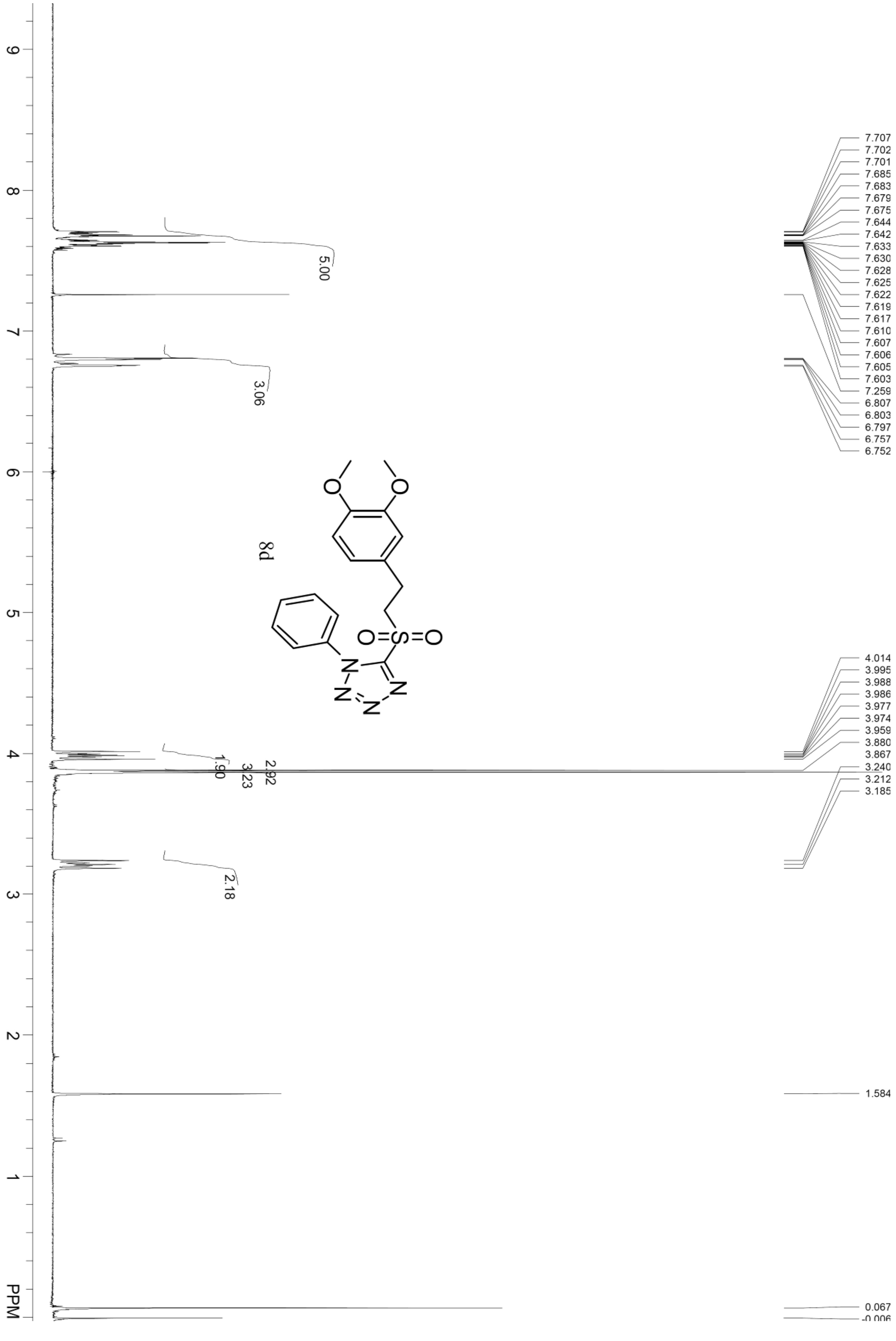
8d
PPM
5.00
3.06
1.90
3.23
2.92
2.18
7.707
7.702
7.701
7.685
7.683
7.679
7.675
7.644
7.642
7.633
7.630
7.628
7.625
7.622
7.619
7.617
7.610
7.607
7.606
7.605
7.603
7.259
6.807
6.803
6.797
6.757
6.752
4.014
3.995
3.988
3.986
3.977
3.974
3.959
3.880
3.867
3.240
3.212
3.185
1.584
0.067
-0.006

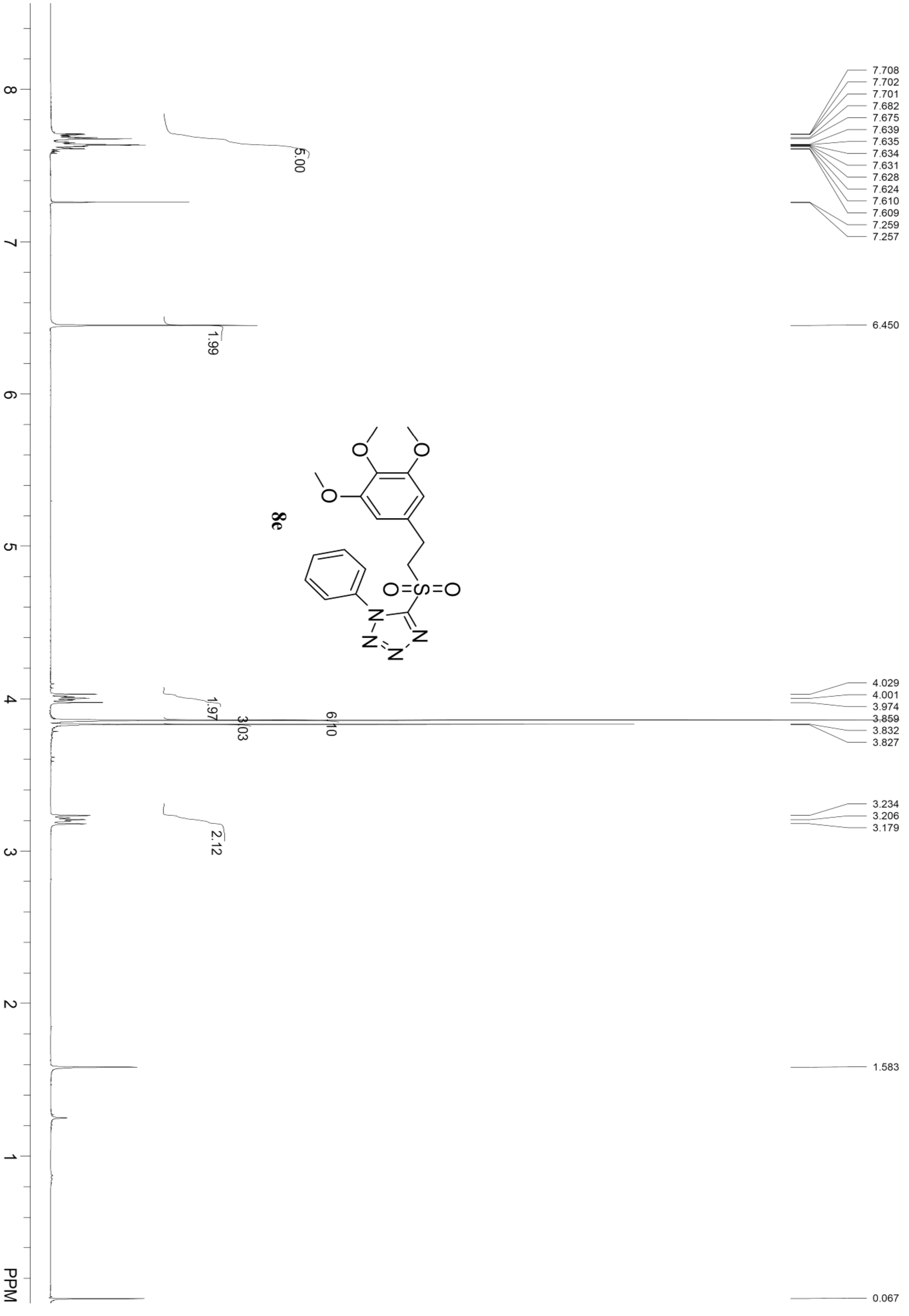
7.708
7.702
7.701
7.682
7.675
7.639
7.635
7.634
7.631
7.628
7.624
7.610
7.609
7.259
7.257
6.450
4.029
4.001
3.974
3.859
3.832
3.827
3.234
3.206
3.179
1.583
0.067
5.00
1.99
1.97
3.03
6.10
2.12
PPM

8e

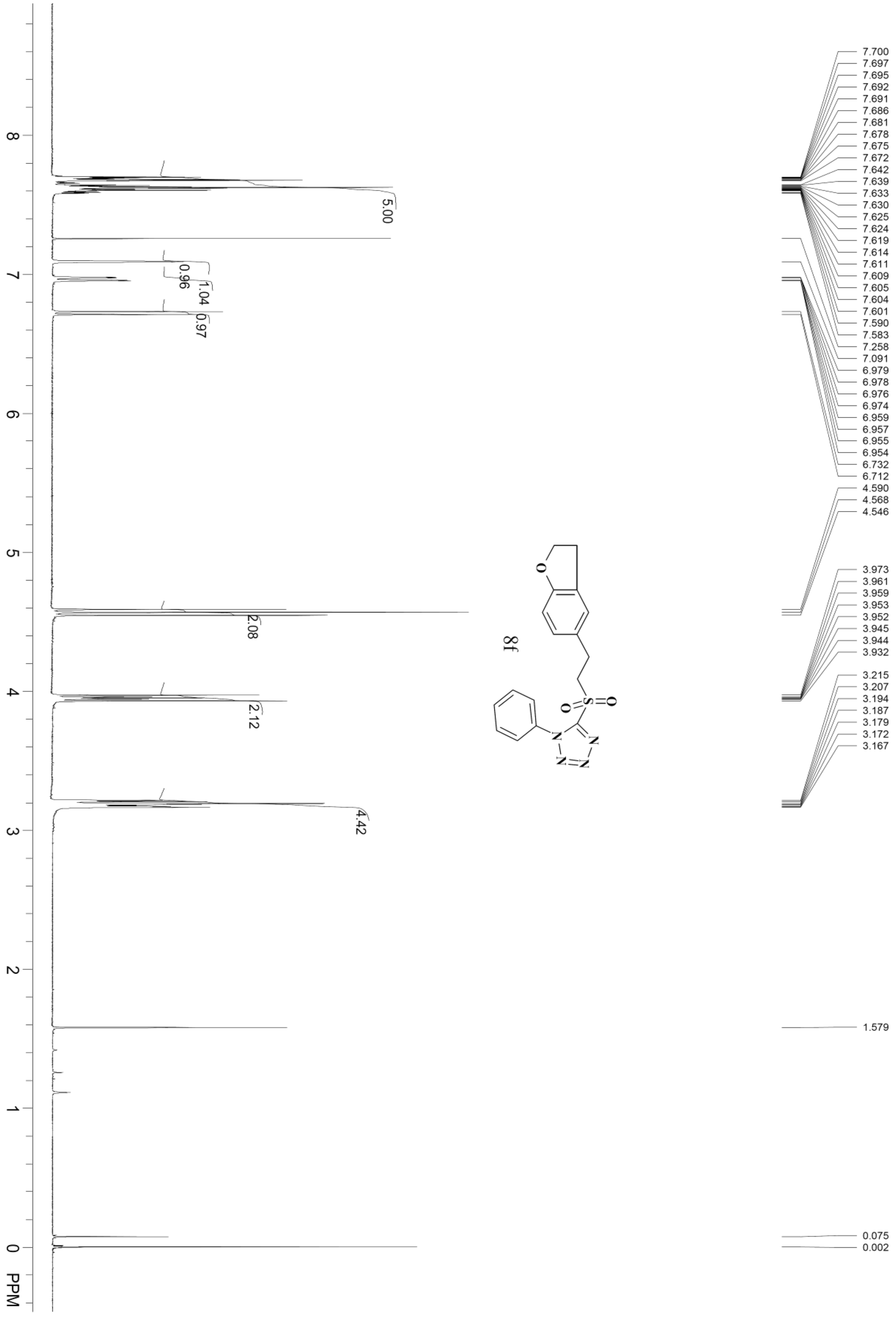
7.700
7.697
7.695
7.692
7.691
7.686
7.681
7.678
7.675
7.672
7.642
7.639
7.633
7.630
7.625
7.624
7.619
7.614
7.611
7.609
7.605
7.604
7.601
7.590
7.583
7.258
7.091
6.979
6.978
6.976
6.974
6.959
6.957
6.955
6.954
6.732
6.712
4.590
4.568
4.546
3.973
3.961
3.959
3.953
3.952
3.945
3.944
3.932
3.215
3.207
3.194
3.187
3.179
3.172
3.167
1.579
0.075
0.002
5.00
0.96
1.04
0.97
2.08
2.12
4.42
8
7
6
5
4
3
2
1
0
PPM
8f

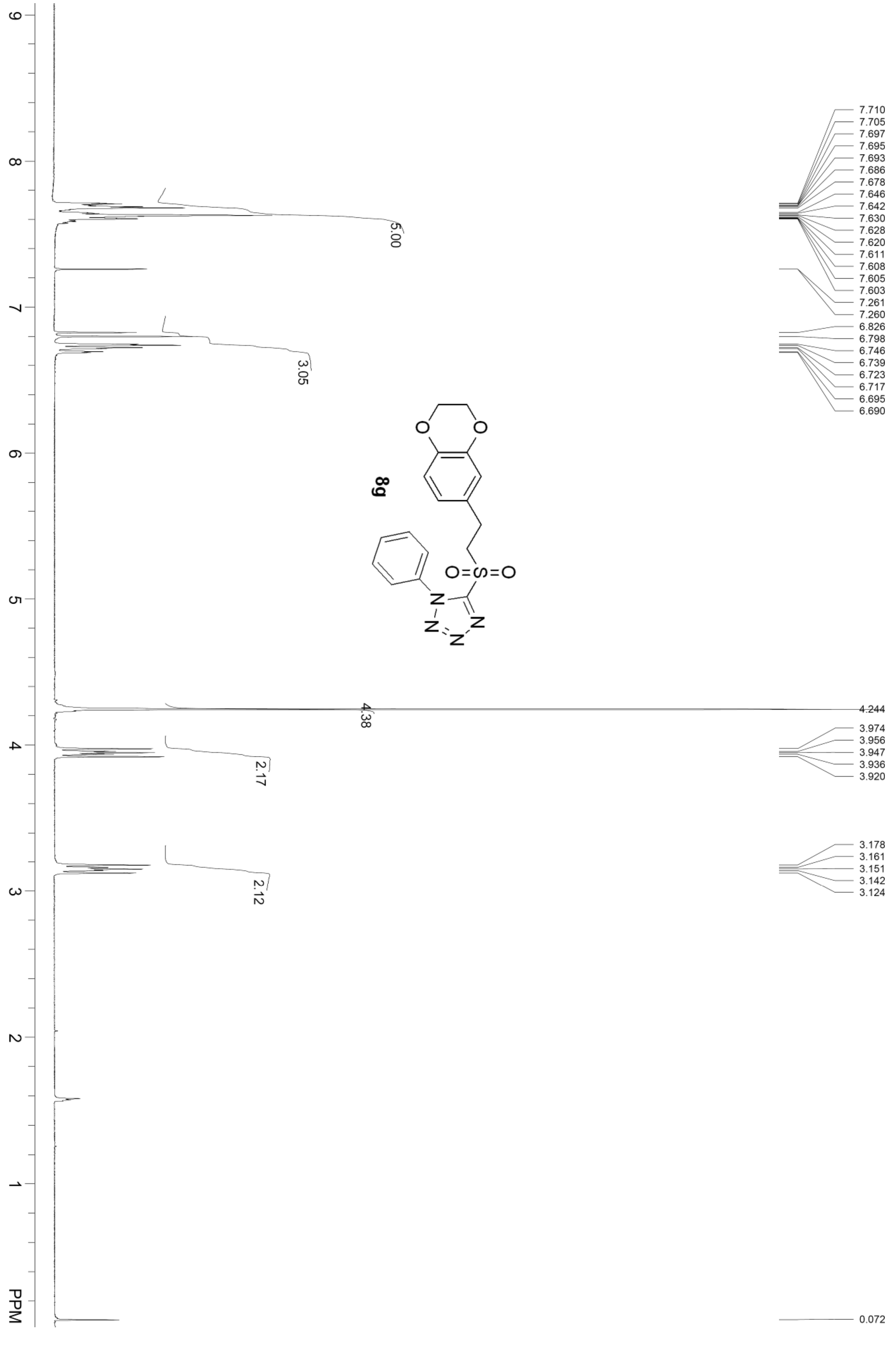
8g
7.710
7.705
7.697
7.695
7.693
7.686
7.678
7.646
7.642
7.630
7.628
7.620
7.611
7.608
7.605
7.603
7.261
7.260
6.826
6.798
6.746
6.739
6.723
6.717
6.695
6.690
4.244
3.974
3.956
3.947
3.936
3.920
3.178
3.161
3.151
3.142
3.124
0.072
5.00
3.05
4.38
2.17
2.12
PPM

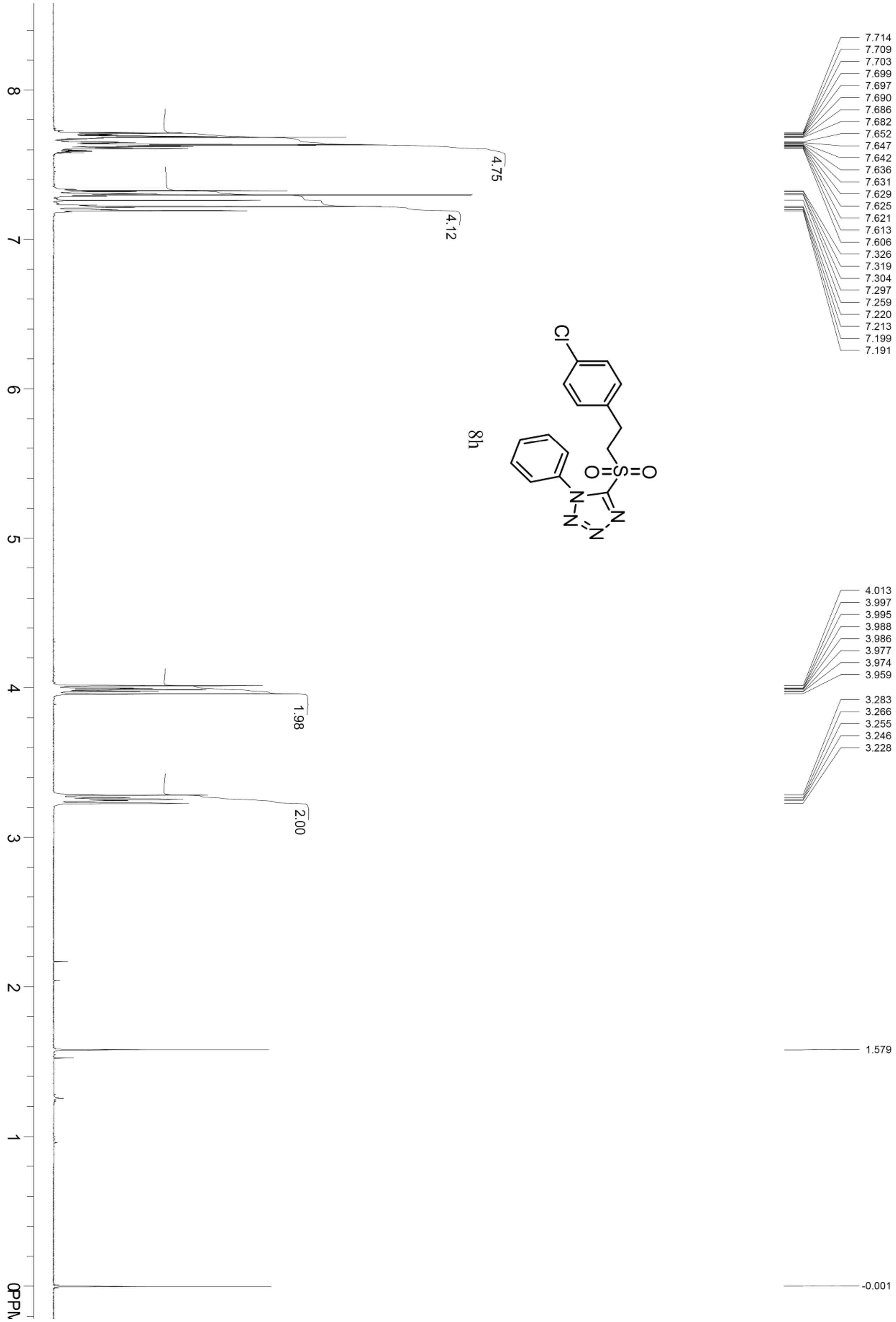
7.714
7.709
7.703
7.699
7.697
7.690
7.686
7.682
7.652
7.647
7.642
7.636
7.631
7.629
7.625
7.621
7.613
7.606
7.326
7.319
7.304
7.297
7.259
7.220
7.213
7.199
7.191
4.75
4.12
Cl
8h
N
S
O
4.013
3.997
3.995
3.988
3.986
3.977
3.974
3.959
1.98
3.283
3.266
3.255
3.246
3.228
2.00
1.579
-0.001
8
7
6
5
4
3
2
1
0PPM

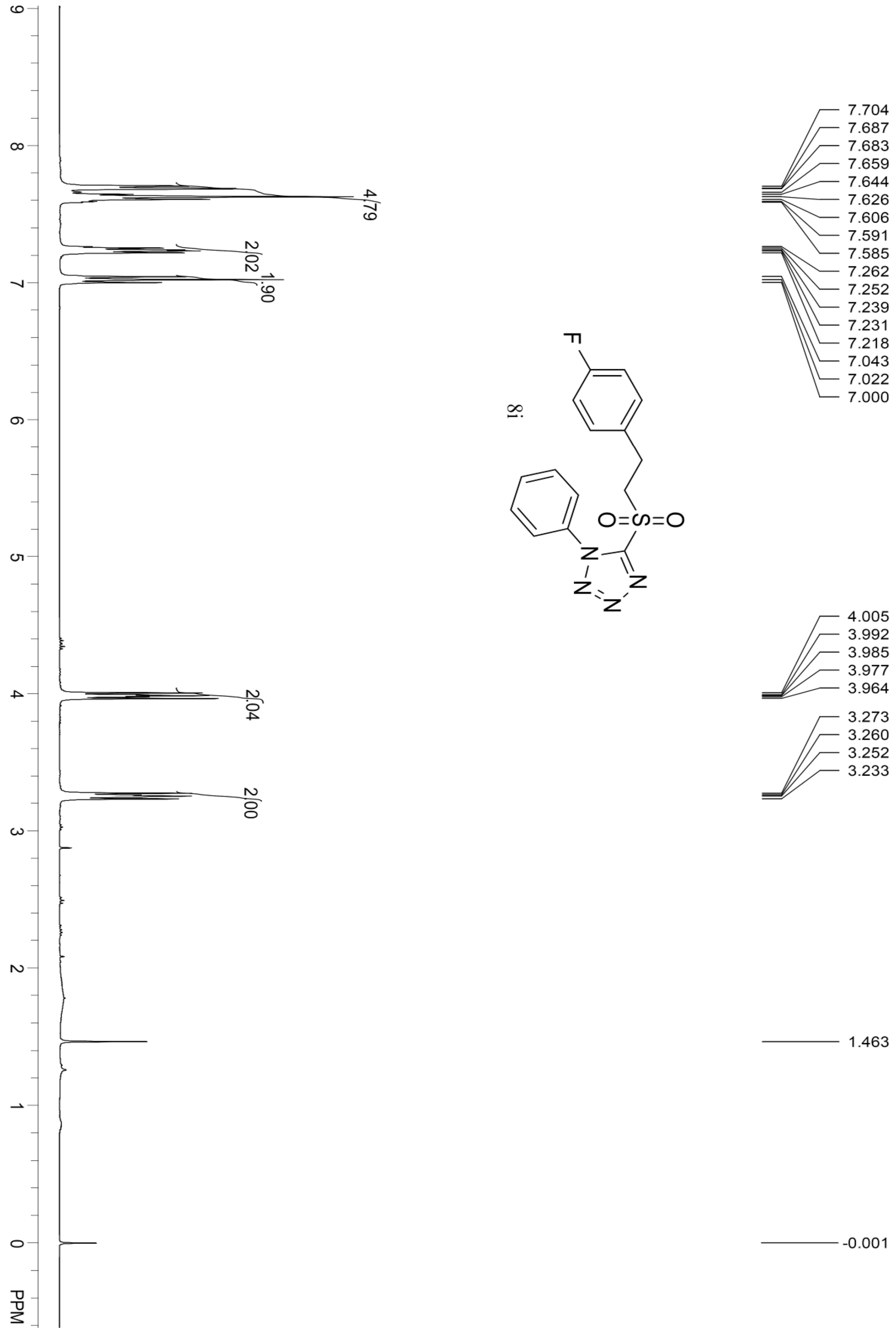
8i
7.704
7.687
7.683
7.659
7.644
7.626
7.606
7.591
7.585
7.262
7.252
7.239
7.231
7.218
7.043
7.022
7.000
4.005
3.992
3.985
3.977
3.964
3.273
3.260
3.252
3.233
1.463
-0.001
4.79
2.02
1.90
2.04
2.00
PPM

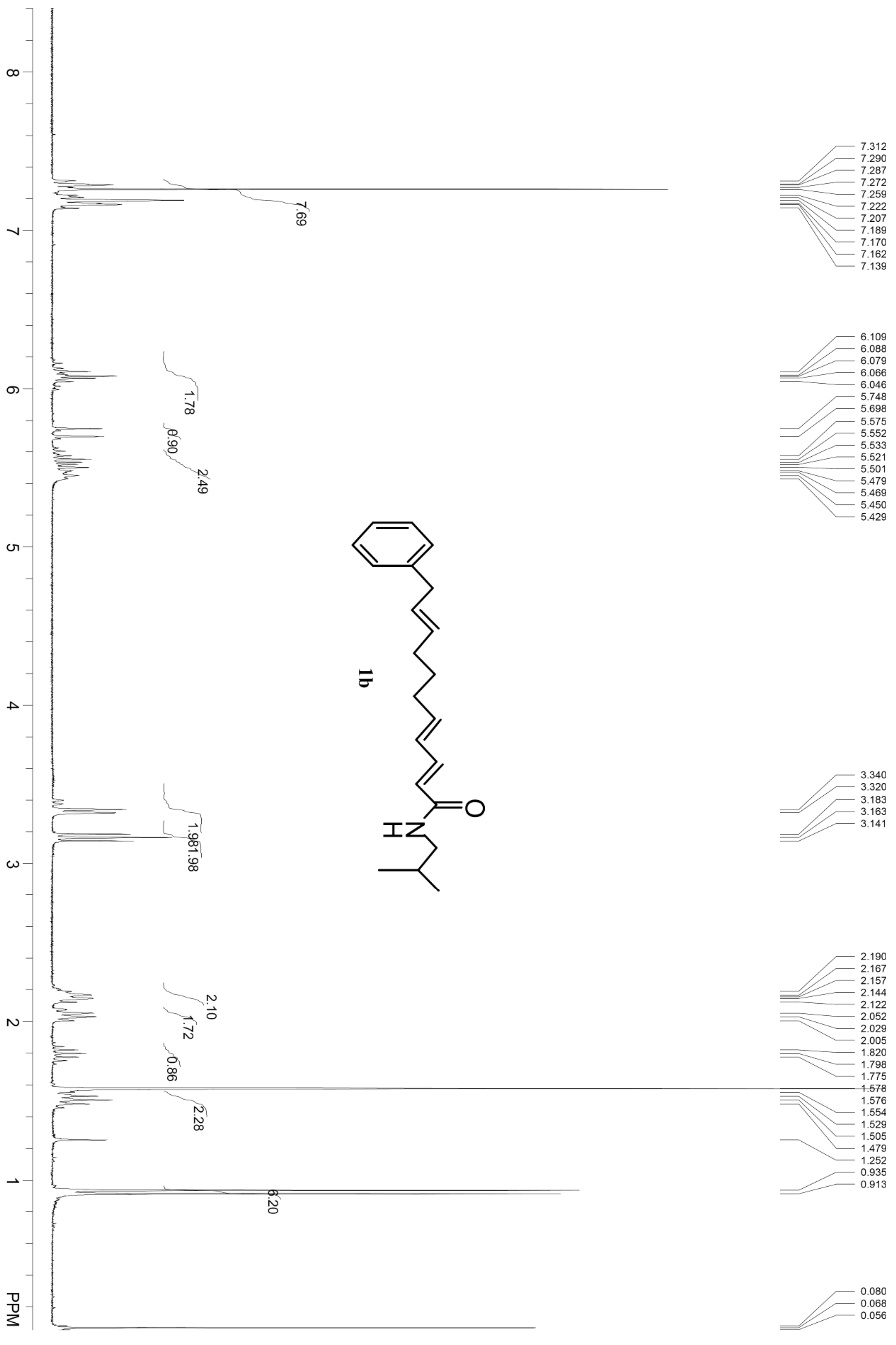
1b

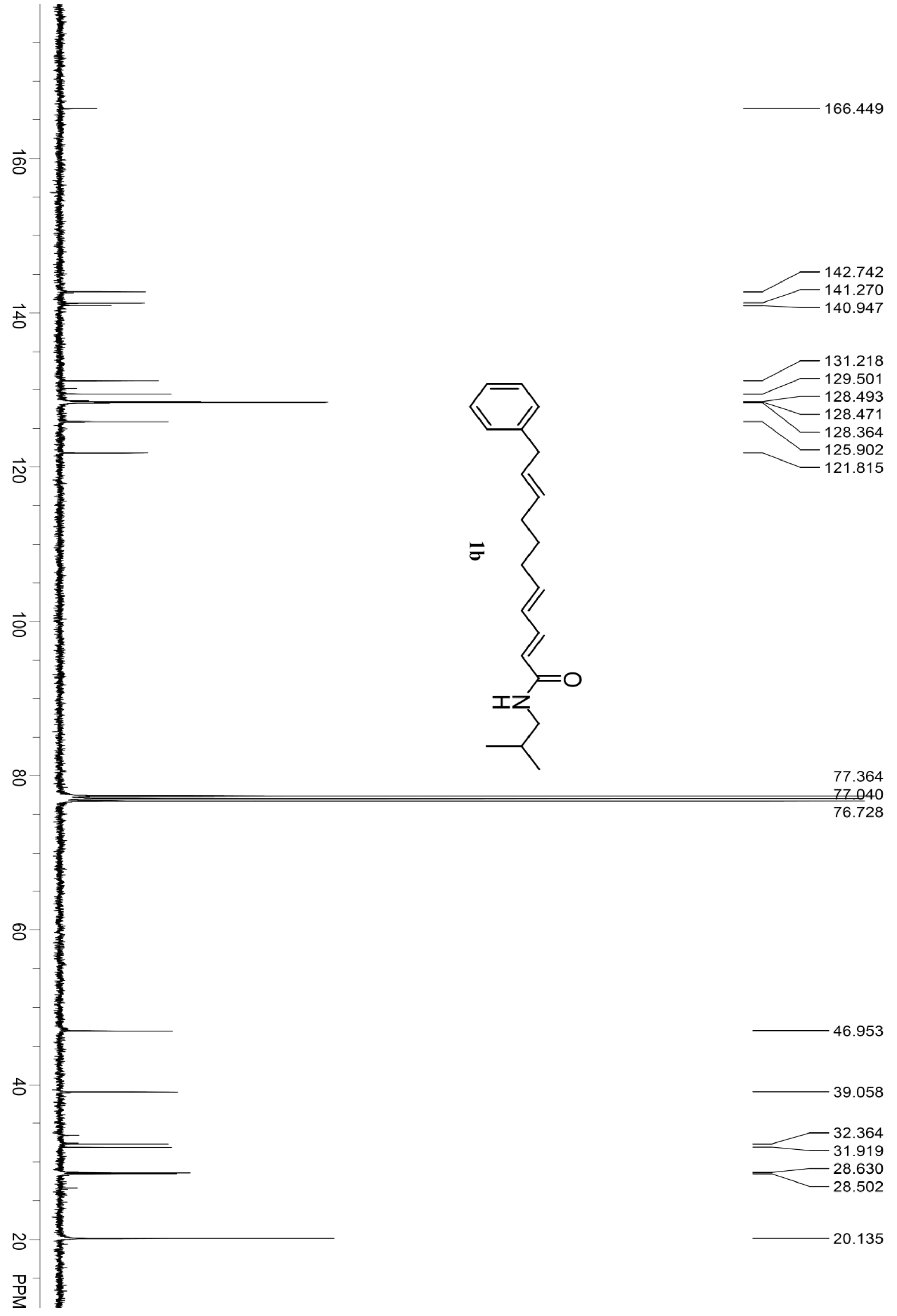
166.449
142.742
141.270
140.947
131.218
129.501
128.493
128.471
128.364
125.902
121.815
77.364
77.040
76.728
46.953
39.058
32.364
31.919
28.630
28.502
20.135
160
140
120
100
80
60
40
20
PPM
1b
O
N
H

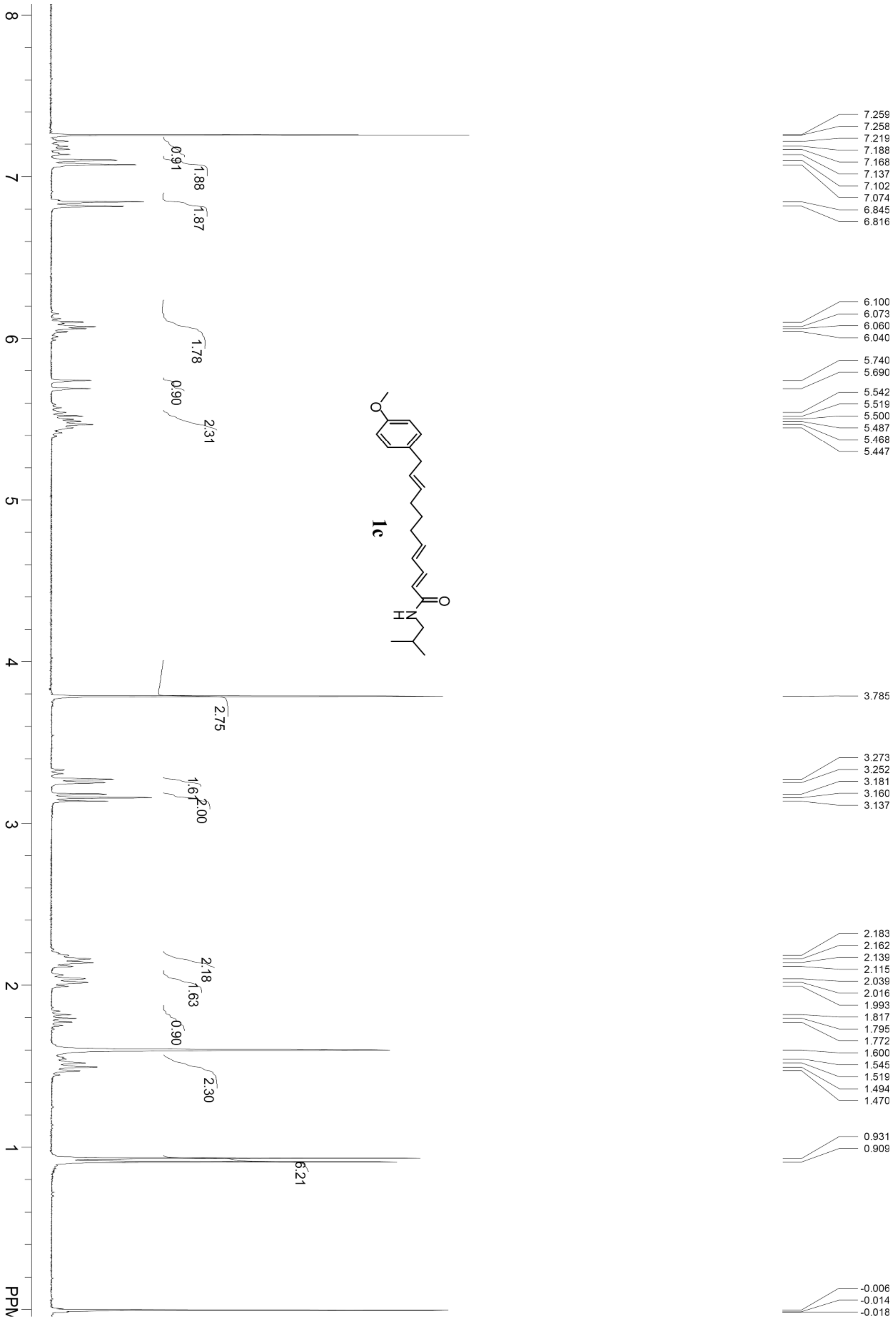
1c
7.259
7.258
7.219
7.188
7.168
7.137
7.102
7.074
6.845
6.816
6.100
6.073
6.060
6.040
5.740
5.690
5.542
5.519
5.500
5.487
5.468
5.447
3.785
3.273
3.252
3.181
3.160
3.137
2.183
2.162
2.139
2.115
2.039
2.016
1.993
1.817
1.795
1.772
1.600
1.545
1.519
1.494
1.470
0.931
0.909
-0.006
-0.014
-0.018
0.91
1.88
1.87
1.78
0.90
2.31
2.75
1.61
2.00
2.18
1.63
0.90
2.30
6.21
PPM

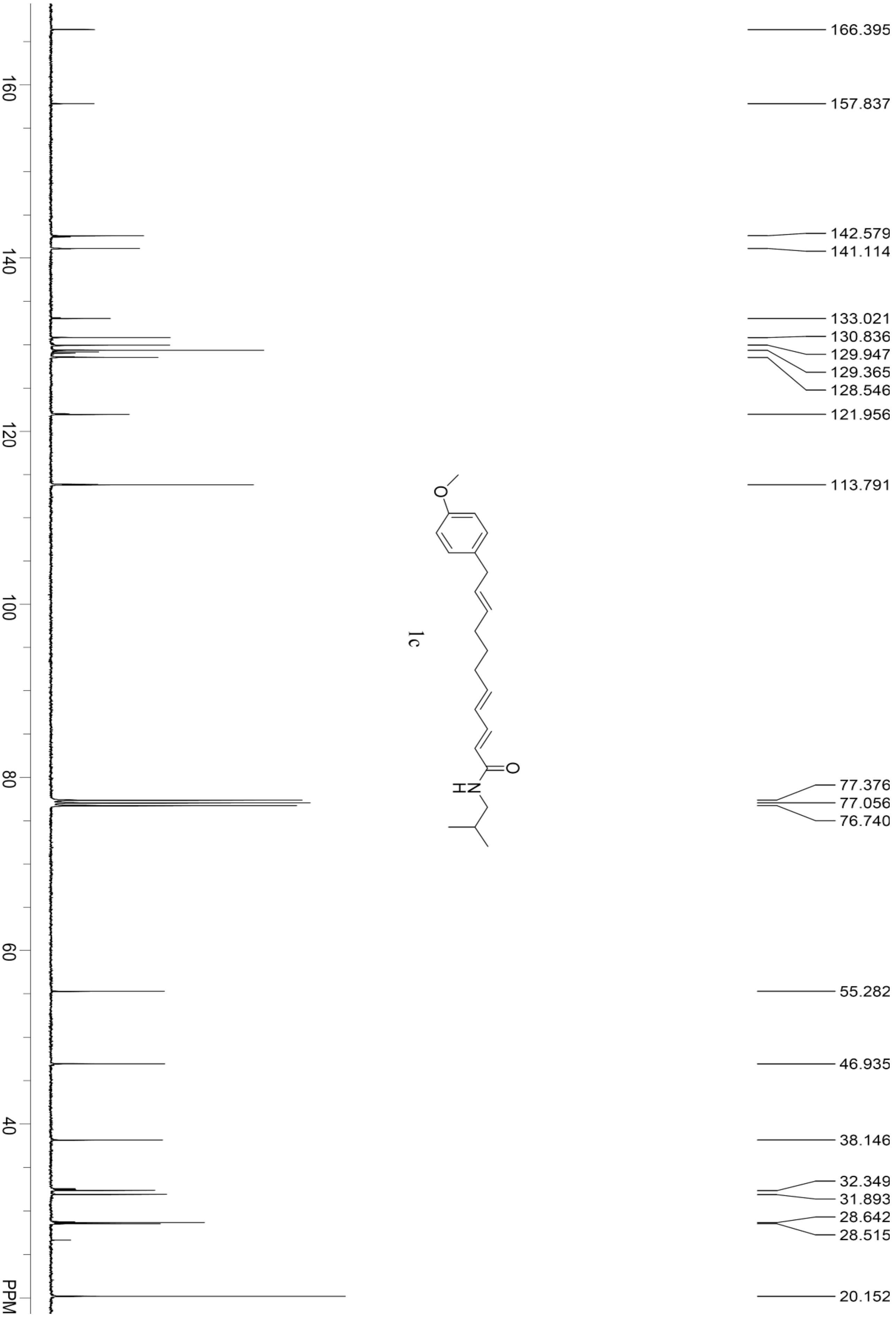
166.395
157.837
142.579
141.114
133.021
130.836
129.947
129.365
128.546
121.956
113.791
77.376
77.056
76.740
55.282
46.935
38.146
32.349
31.893
28.642
28.515
20.152
160
140
120
100
80
60
40
PPM
1c

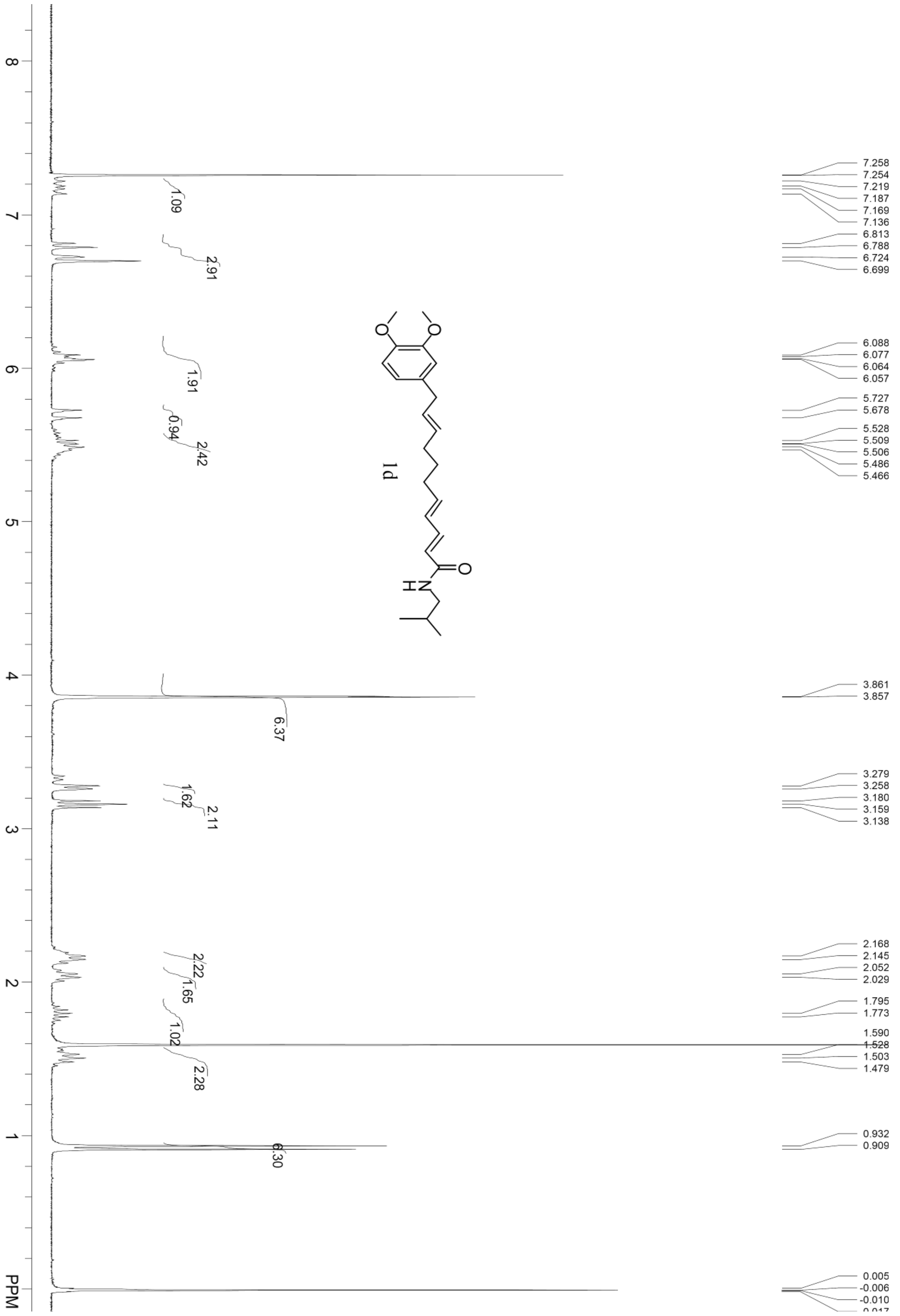
7.258
7.254
7.219
7.187
7.169
7.136
6.813
6.788
6.724
6.699
6.088
6.077
6.064
6.057
5.727
5.678
5.528
5.509
5.506
5.486
5.466
3.861
3.857
3.279
3.258
3.180
3.159
3.138
2.168
2.145
2.052
2.029
1.795
1.773
1.590
1.528
1.503
1.479
0.932
0.909
0.005
-0.006
-0.010
1d
PPM

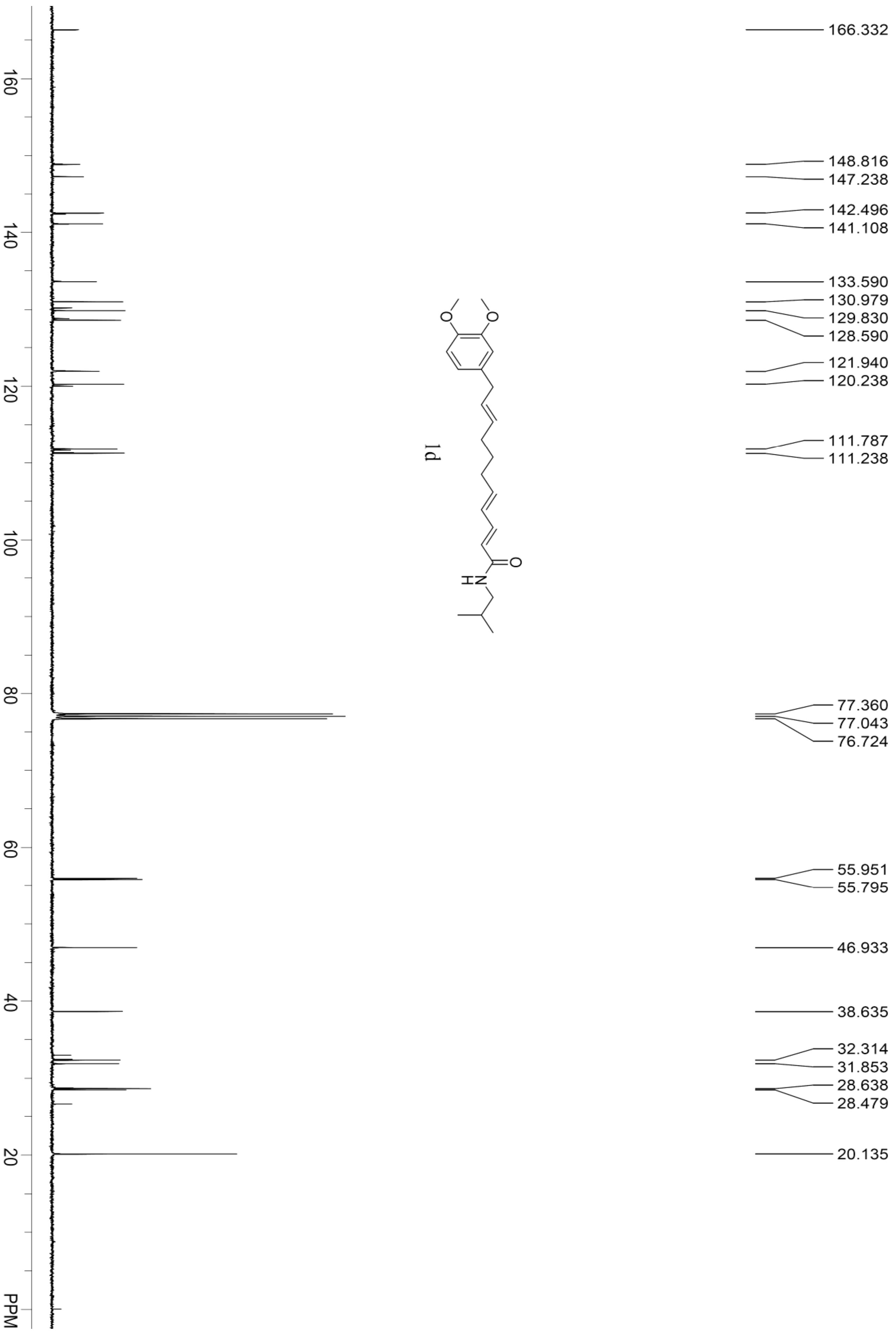
166.332
148.816
147.238
142.496
141.108
133.590
130.979
129.830
128.590
121.940
120.238
111.787
111.238
77.360
77.043
76.724
55.951
55.795
46.933
38.635
32.314
31.853
28.638
28.479
20.135
1d
160
140
120
100
80
60
40
20
PPM

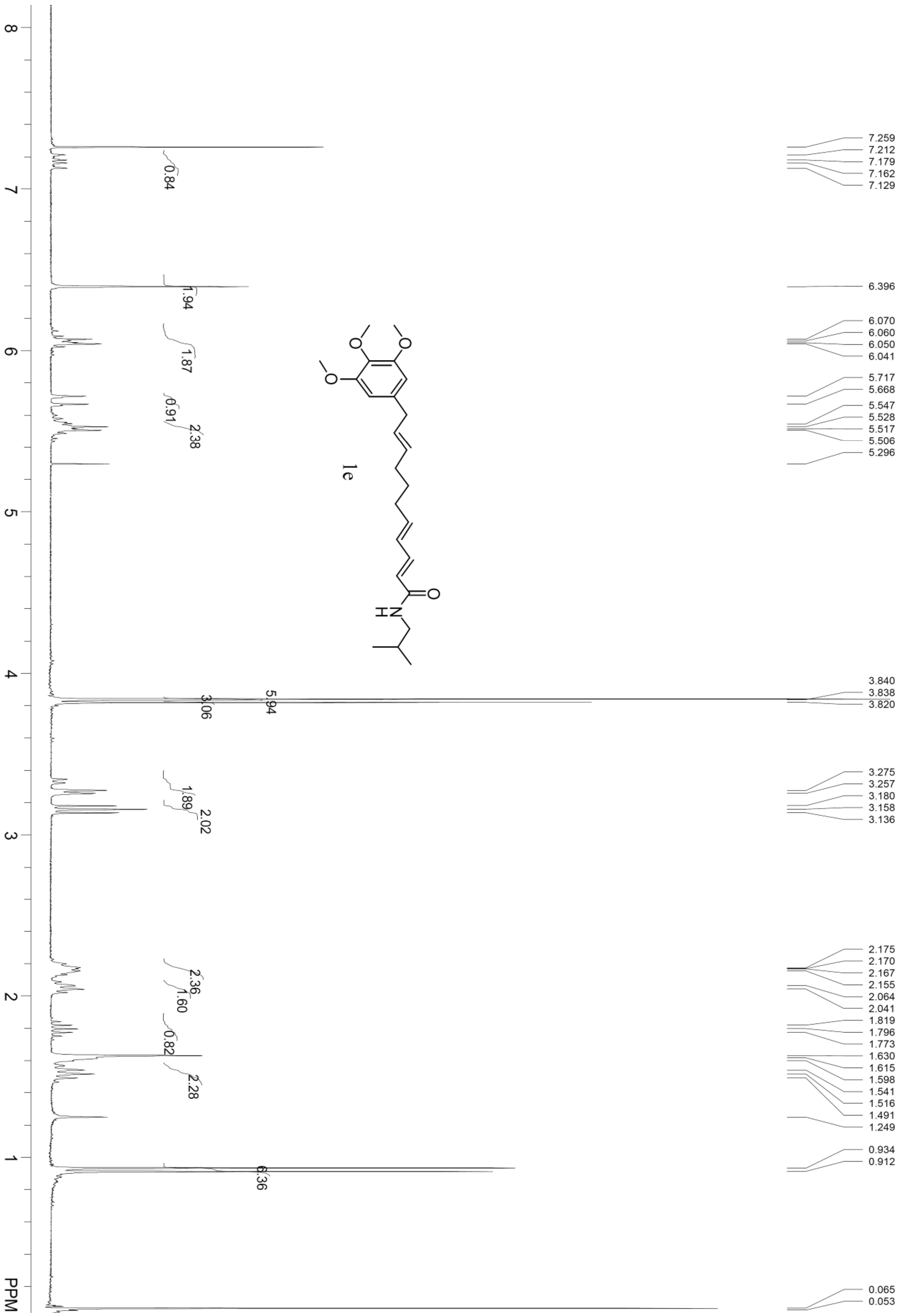
1e
PPM
7.259
7.212
7.179
7.162
7.129
6.396
6.070
6.060
6.050
6.041
5.717
5.668
5.547
5.528
5.517
5.506
5.296
3.840
3.838
3.820
3.275
3.257
3.180
3.158
3.136
2.175
2.170
2.167
2.155
2.064
2.041
1.819
1.796
1.773
1.630
1.615
1.598
1.541
1.516
1.491
1.249
0.934
0.912
0.065
0.053

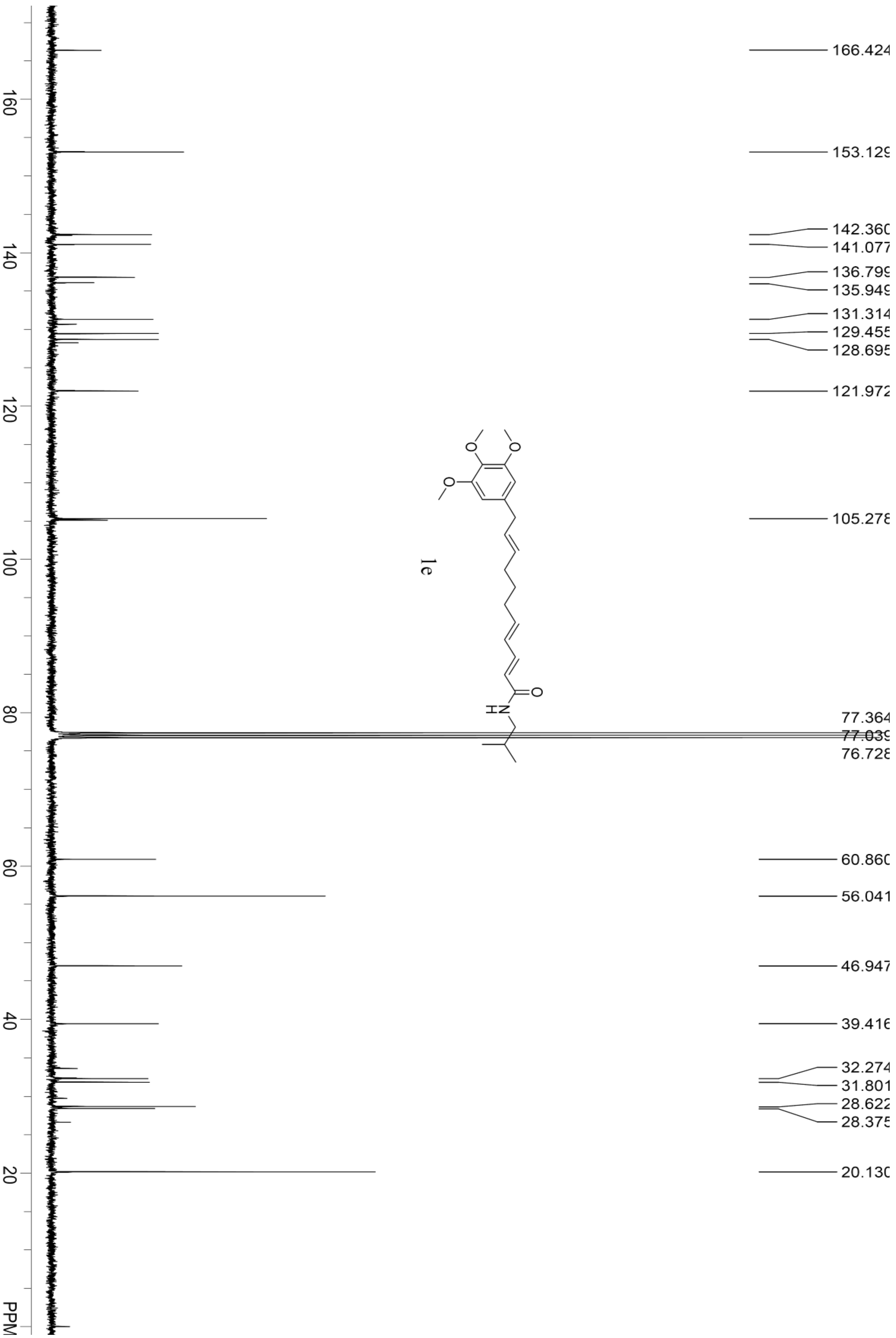
166.424
153.129
142.360
141.077
136.799
135.949
131.314
129.455
128.695
121.972
105.278
1e
77.364
77.039
76.728
60.860
56.041
46.947
39.416
32.274
31.801
28.622
28.375
20.130
160
140
120
100
80
60
40
20
PPM

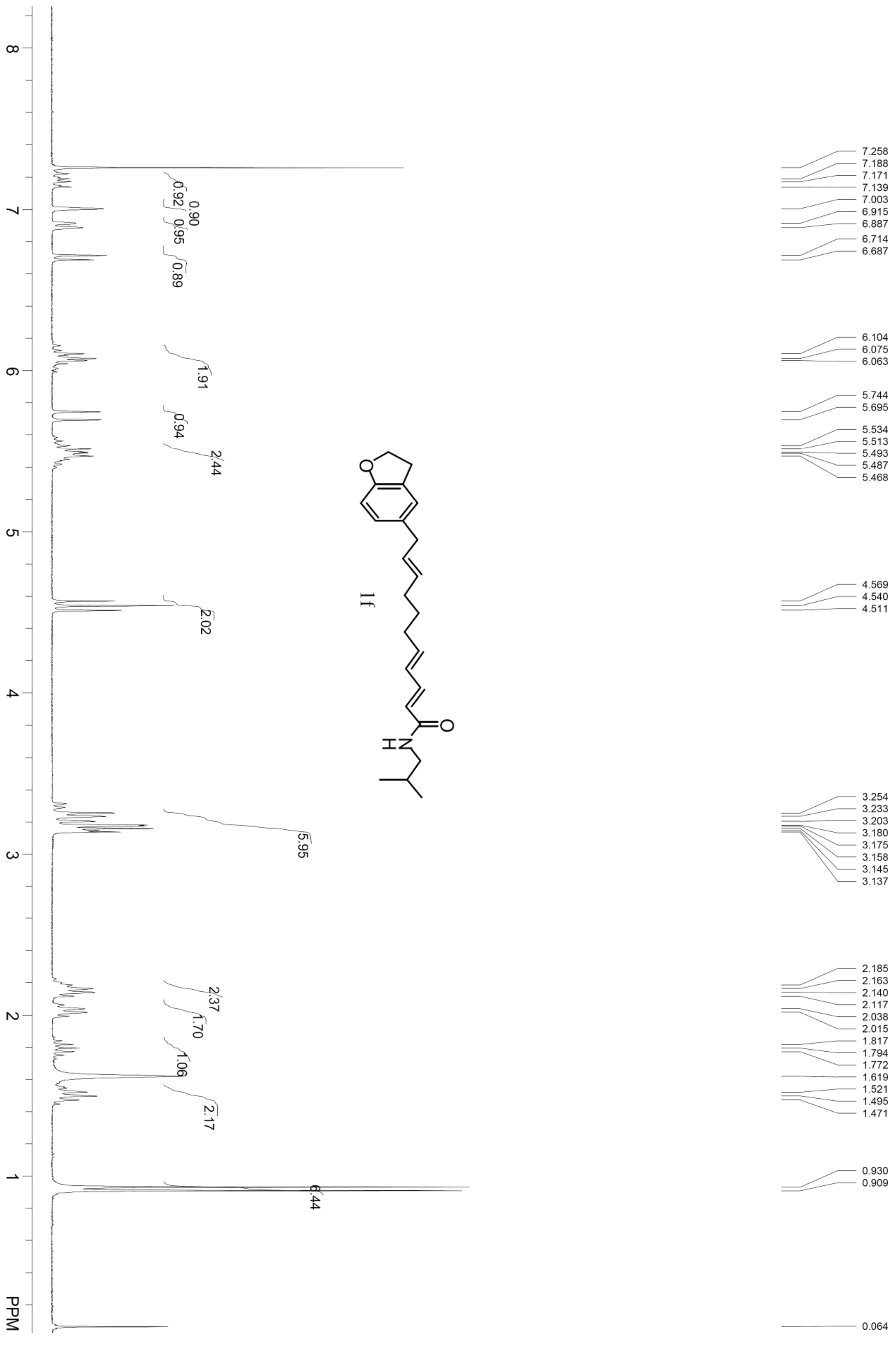
7.258
7.188
7.171
7.139
7.003
6.915
6.887
6.714
6.687
6.104
6.075
6.063
5.744
5.695
5.534
5.513
5.493
5.487
5.468
4.569
4.540
4.511
3.254
3.233
3.203
3.180
3.175
3.158
3.145
3.137
2.185
2.163
2.140
2.117
2.038
2.015
1.817
1.794
1.772
1.619
1.521
1.495
1.471
0.930
0.909
0.064
1f
PPM

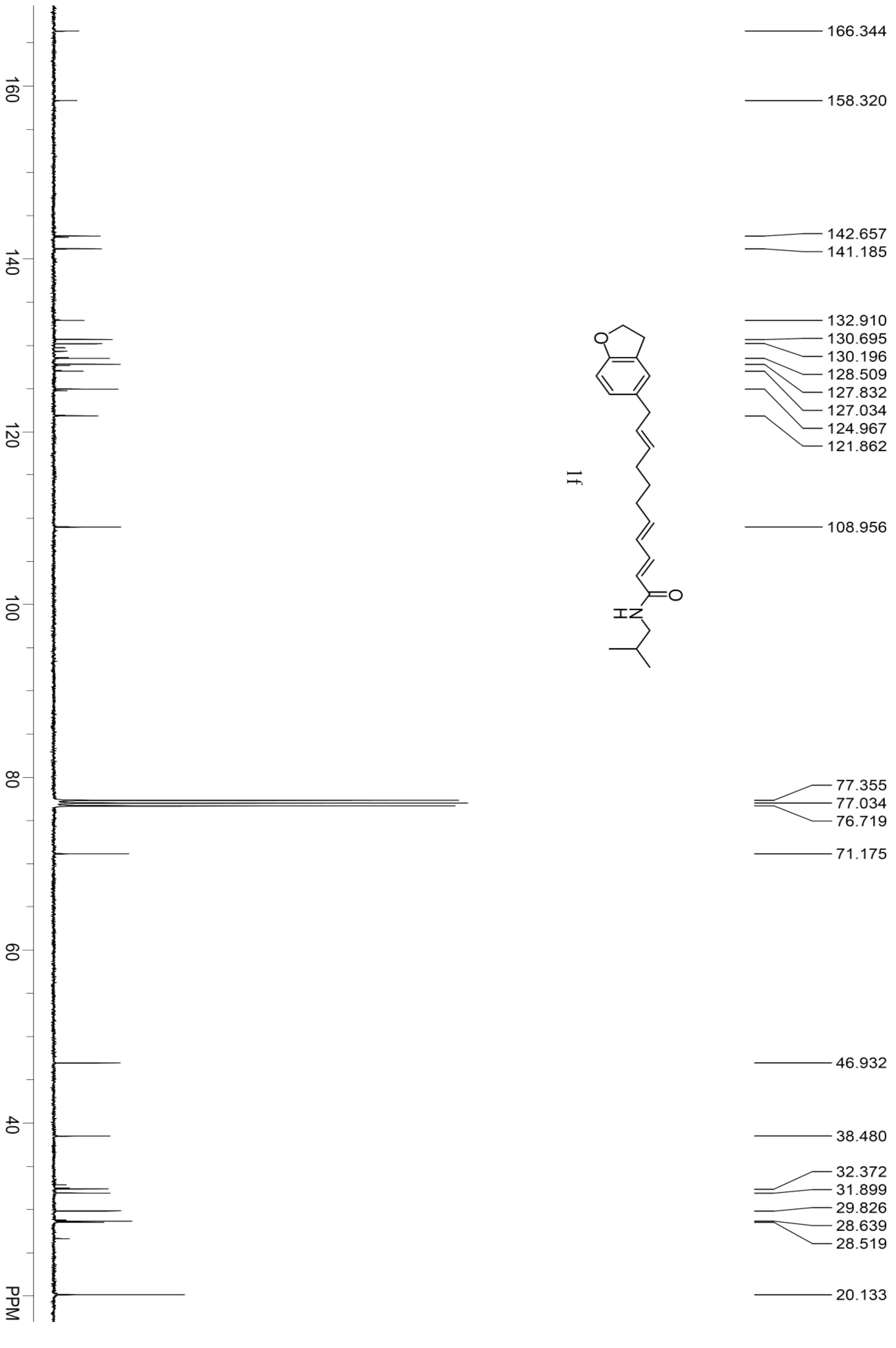
166.344
158.320
142.657
141.185
132.910
130.695
130.196
128.509
127.832
127.034
124.967
121.862
108.956
77.355
77.034
76.719
71.175
46.932
38.480
32.372
31.899
29.826
28.639
28.519
20.133
1f
160
140
120
100
80
60
40
PPM

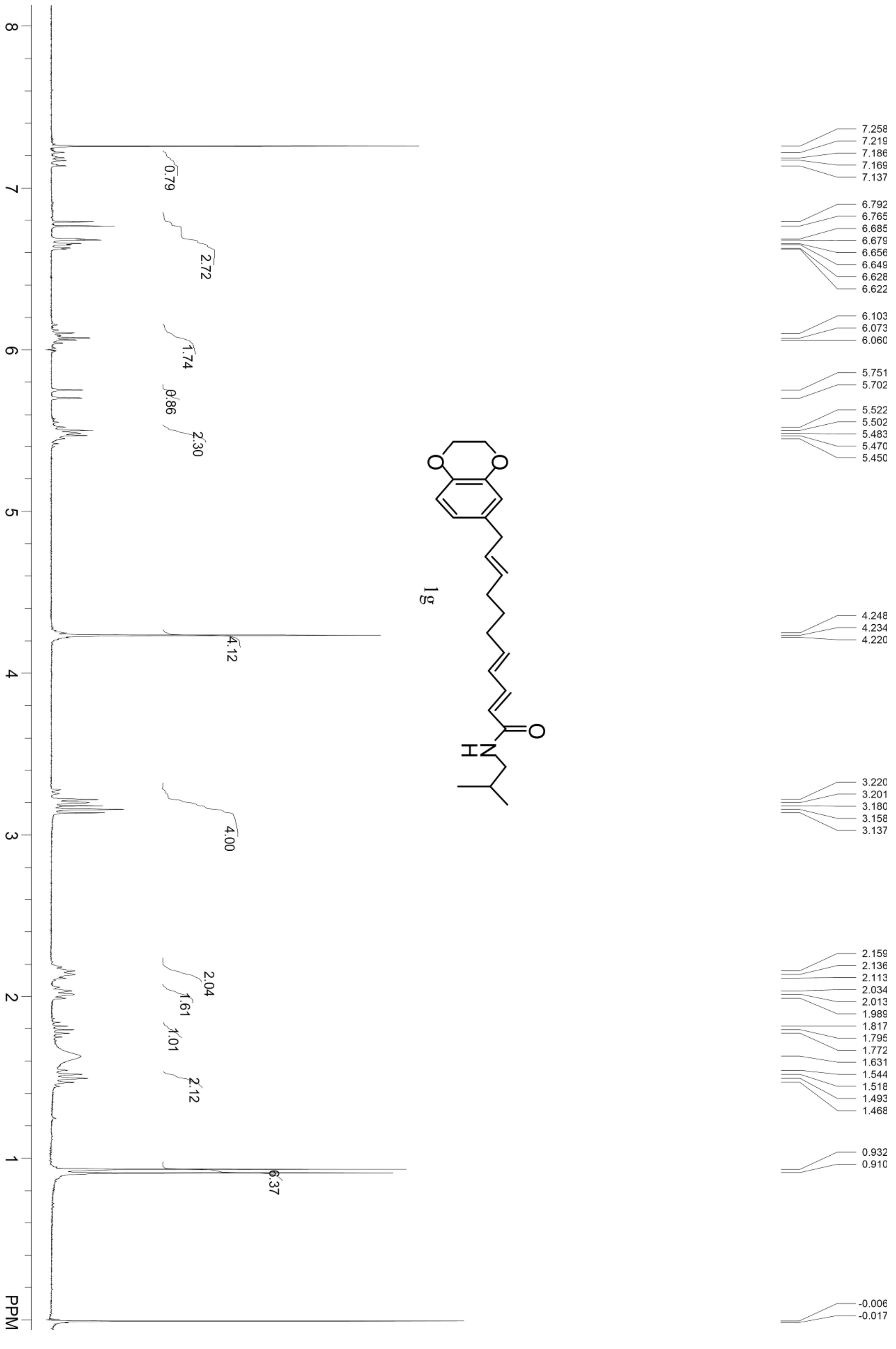
1g
PPM
7.258
7.219
7.186
7.169
7.137
6.792
6.765
6.685
6.679
6.656
6.649
6.628
6.622
6.103
6.073
6.060
5.751
5.702
5.522
5.502
5.483
5.470
5.450
4.248
4.234
4.220
3.220
3.201
3.180
3.158
3.137
2.159
2.136
2.113
2.034
2.013
1.989
1.817
1.795
1.772
1.631
1.544
1.518
1.493
1.468
0.932
0.910
-0.006
-0.017

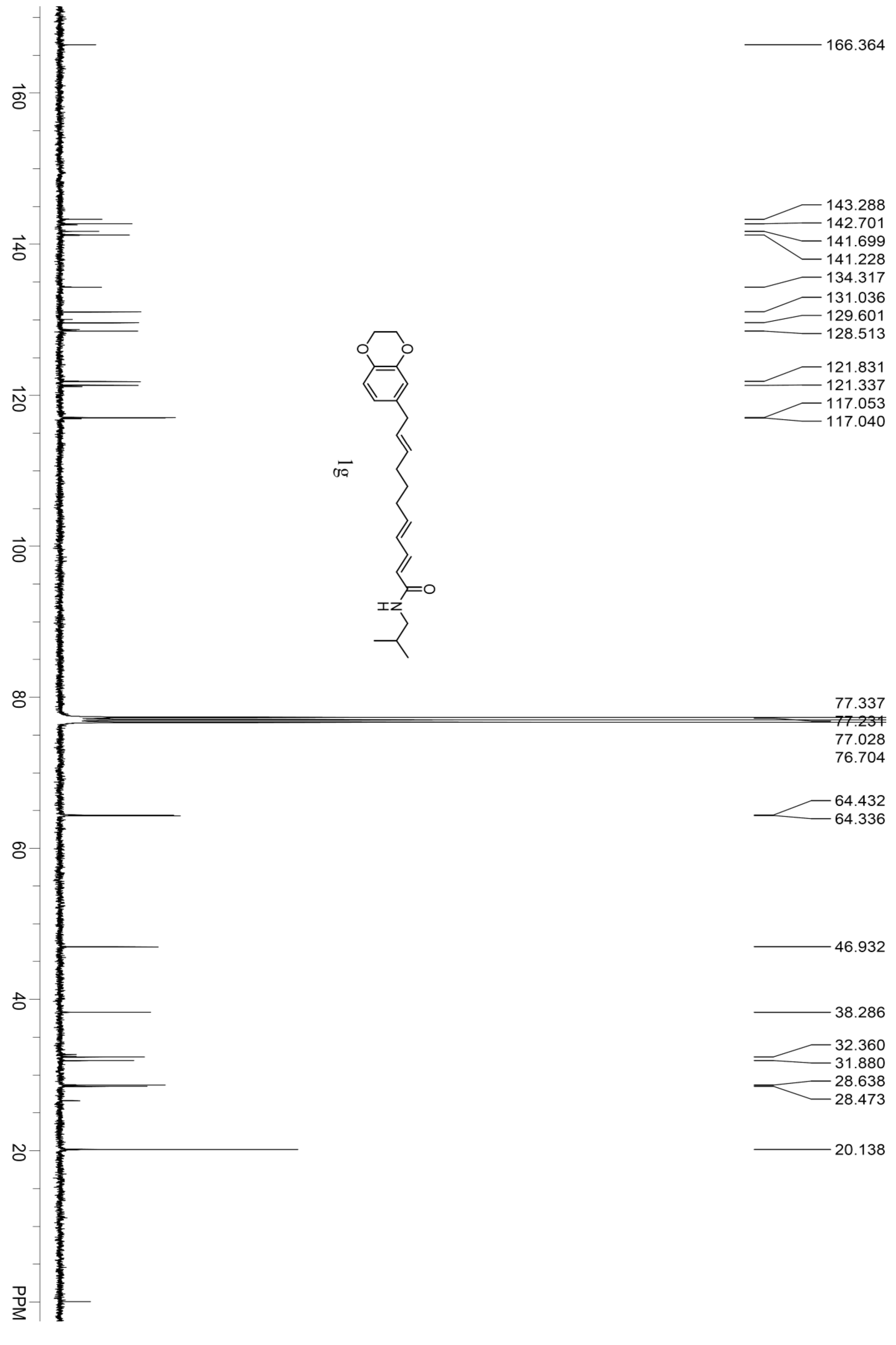
166.364
143.288
142.701
141.699
141.228
134.317
131.036
129.601
128.513
121.831
121.337
117.053
117.040
77.337
77.231
77.028
76.704
64.432
64.336
46.932
38.286
32.360
31.880
28.638
28.473
20.138
1g
160
140
120
100
80
60
40
20
PPM

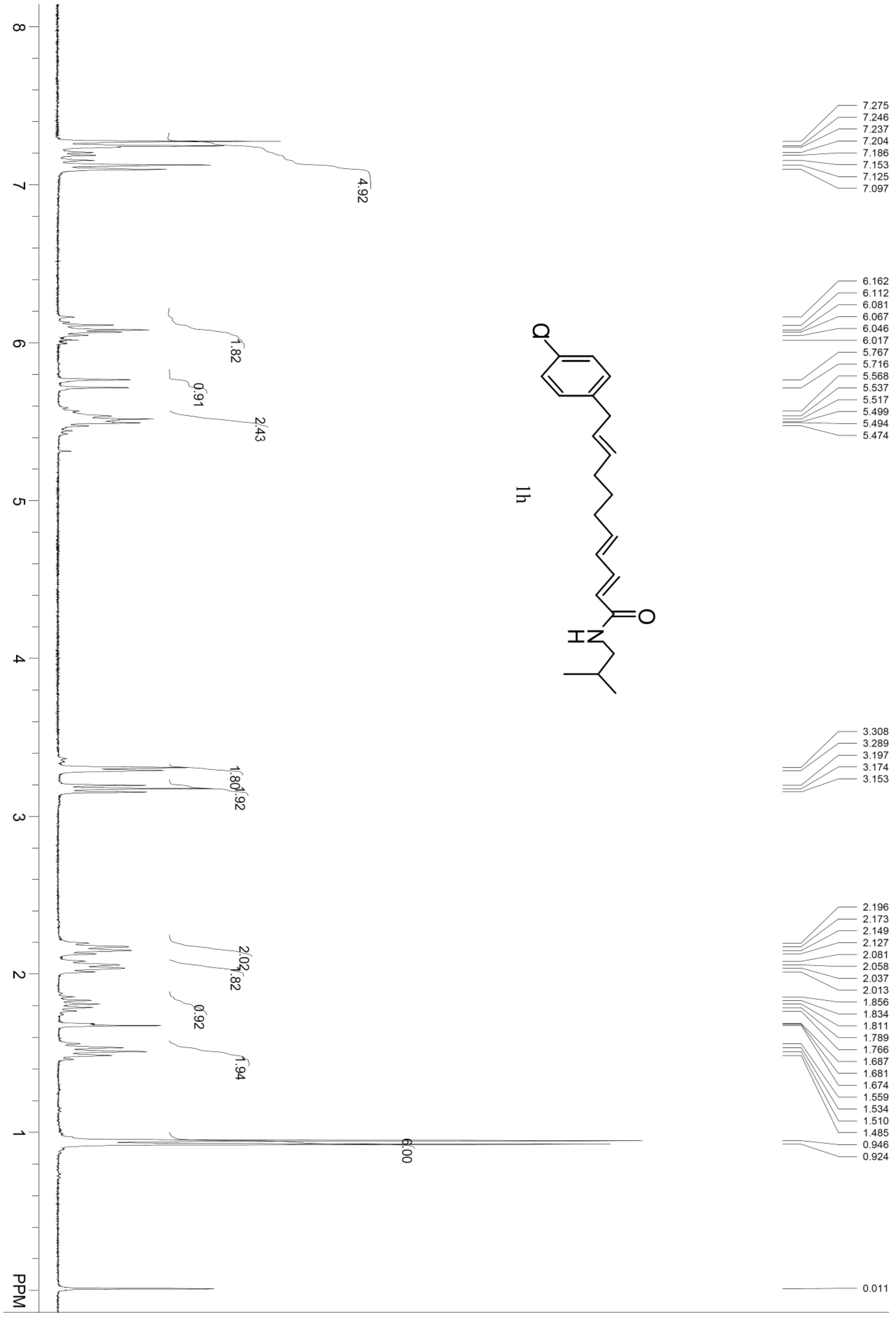
7.275
7.246
7.237
7.204
7.186
7.153
7.125
7.097
6.162
6.112
6.081
6.067
6.046
6.017
5.767
5.716
5.568
5.537
5.517
5.499
5.494
5.474
3.308
3.289
3.197
3.174
3.153
2.196
2.173
2.149
2.127
2.081
2.058
2.037
2.013
1.856
1.834
1.811
1.789
1.766
1.687
1.681
1.674
1.559
1.534
1.510
1.485
0.946
0.924
0.011
4.92
1.82
0.91
2.43
1.80
1.92
2.02
1.82
0.92
1.94
6.00
Cl
O
N
H
1h
PPM
8
7
6
5
4
3
2
1

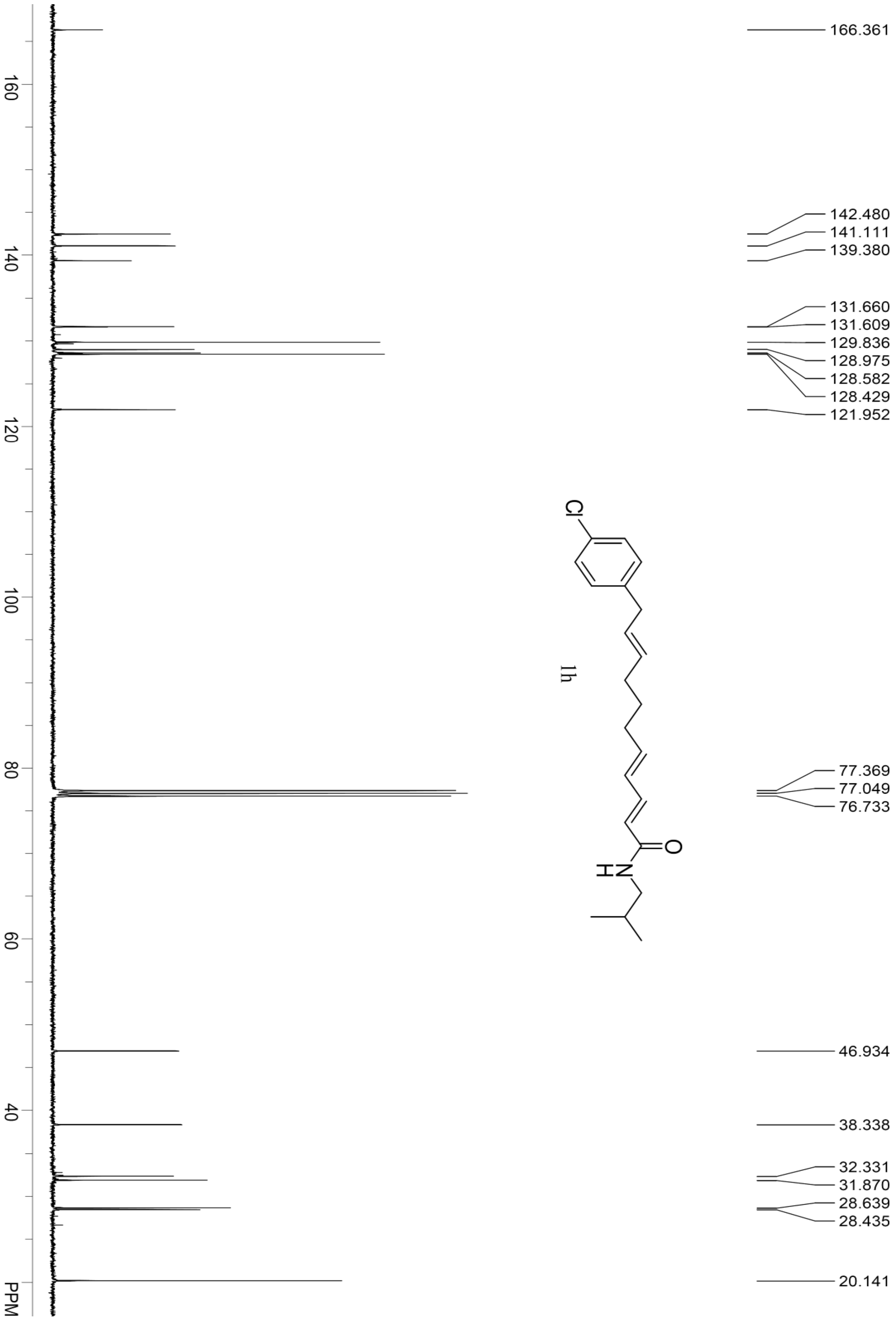
166.361
142.480
141.111
139.380
131.660
131.609
129.836
128.975
128.582
128.429
121.952
77.369
77.049
76.733
46.934
38.338
32.331
31.870
28.639
28.435
20.141
160
140
120
100
80
60
40
PPM
Cl
O
N
H
1h

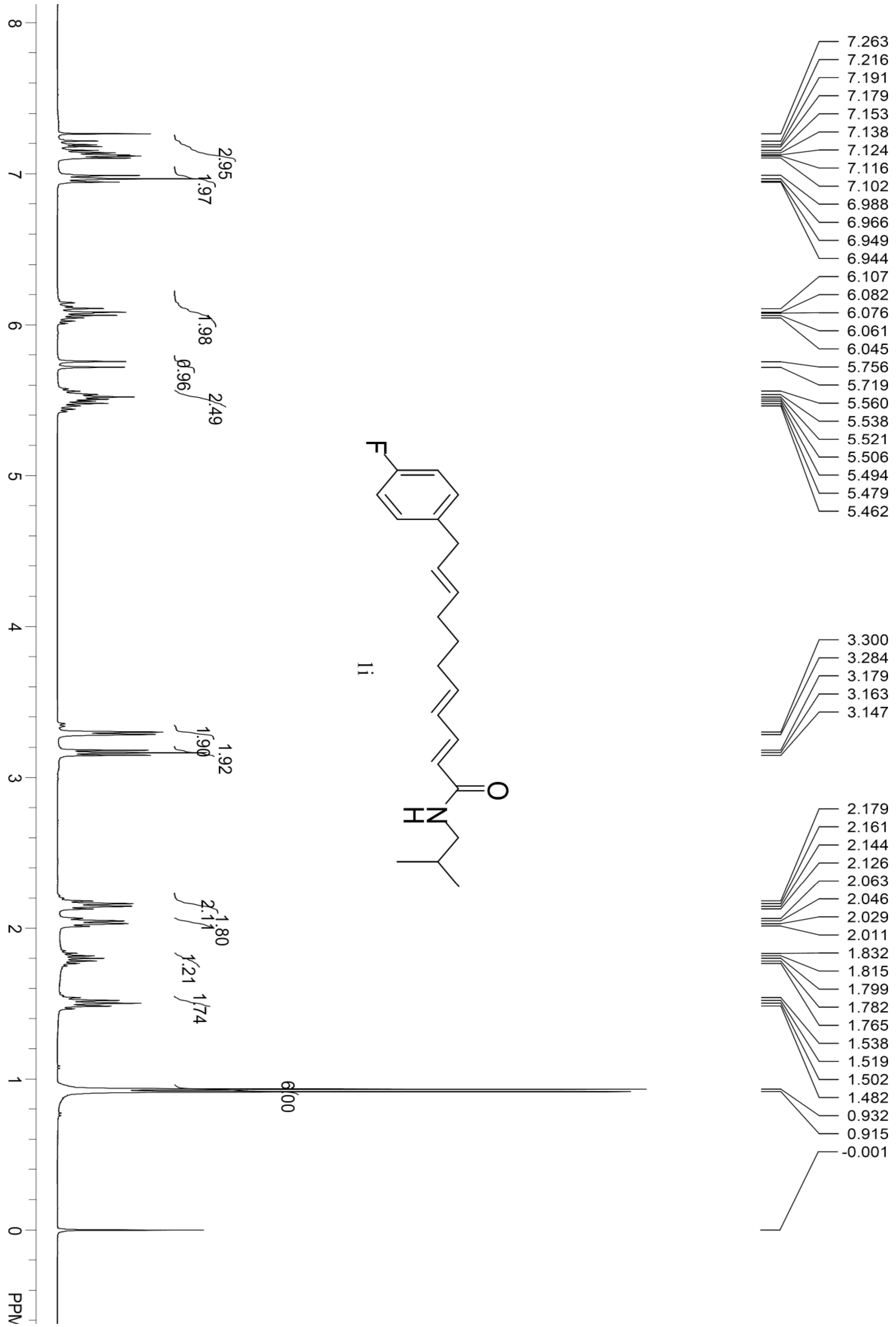
1i
7.263
7.216
7.191
7.179
7.153
7.138
7.124
7.116
7.102
6.988
6.966
6.949
6.944
6.107
6.082
6.076
6.061
6.045
5.756
5.719
5.560
5.538
5.521
5.506
5.494
5.479
5.462
3.300
3.284
3.179
3.163
3.147
2.179
2.161
2.144
2.126
2.063
2.046
2.029
2.011
1.832
1.815
1.799
1.782
1.765
1.538
1.519
1.502
1.482
0.932
0.915
-0.001
PPM

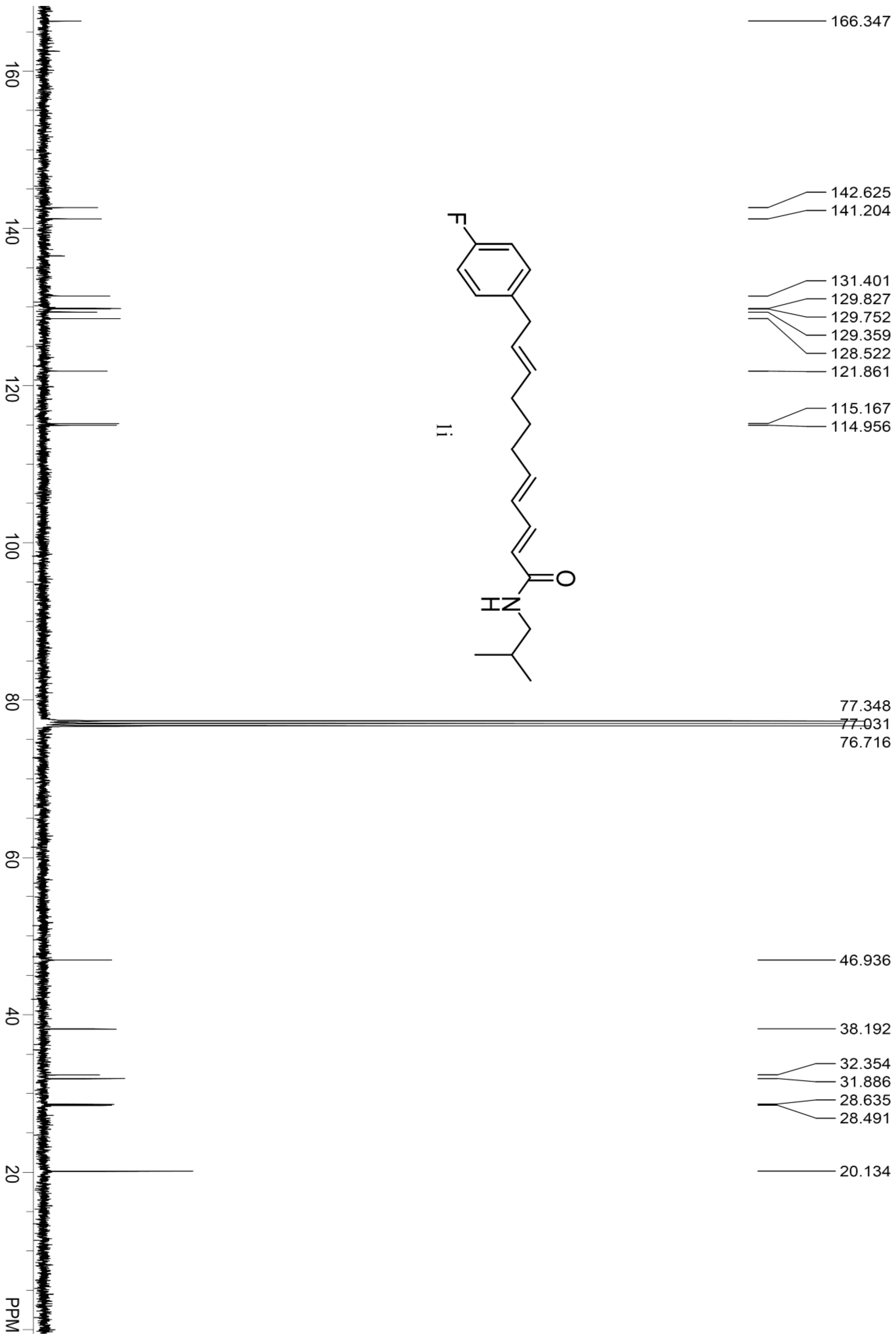
166.347
142.625
141.204
131.401
129.827
129.752
129.359
128.522
121.861
115.167
114.956
77.348
77.031
76.716
46.936
38.192
32.354
31.886
28.635
28.491
20.134
1i
PPM
